# Supplementary material for: Rapid emergence of extensively drug-resistant Shigella sonnei in France
Source: Nat Commun. 2023 Jan 28;14:462. doi: 10.1038/s41467-023-36222-8 (PMC9883819; doi:10.1038/s41467-023-36222-8)
Supplement: Supplementary file 1 — Supplementary Information [file 41467_2023_36222_MOESM1_ESM.pdf]

Supplementary Information

Recent and rapid emergence of extensively drug-resistant *Shigella sonnei* in France

Sophie Lefèvre, Elisabeth Njamkepo, Sarah Feldman, Corinne Ruckly, Isabelle Carle, Monique Lejay-Collin, Laëtitia Fabre, Iman Yassine, Lise Frézal, Maria Pardos de la Gandara, Arnaud Fontanet, François-Xavier Weill

Supplementary Figure 1. Circular map and comparative analysis of the ~ 8 kb MDR plasmids present in our XDR *S. sonnei* isolates

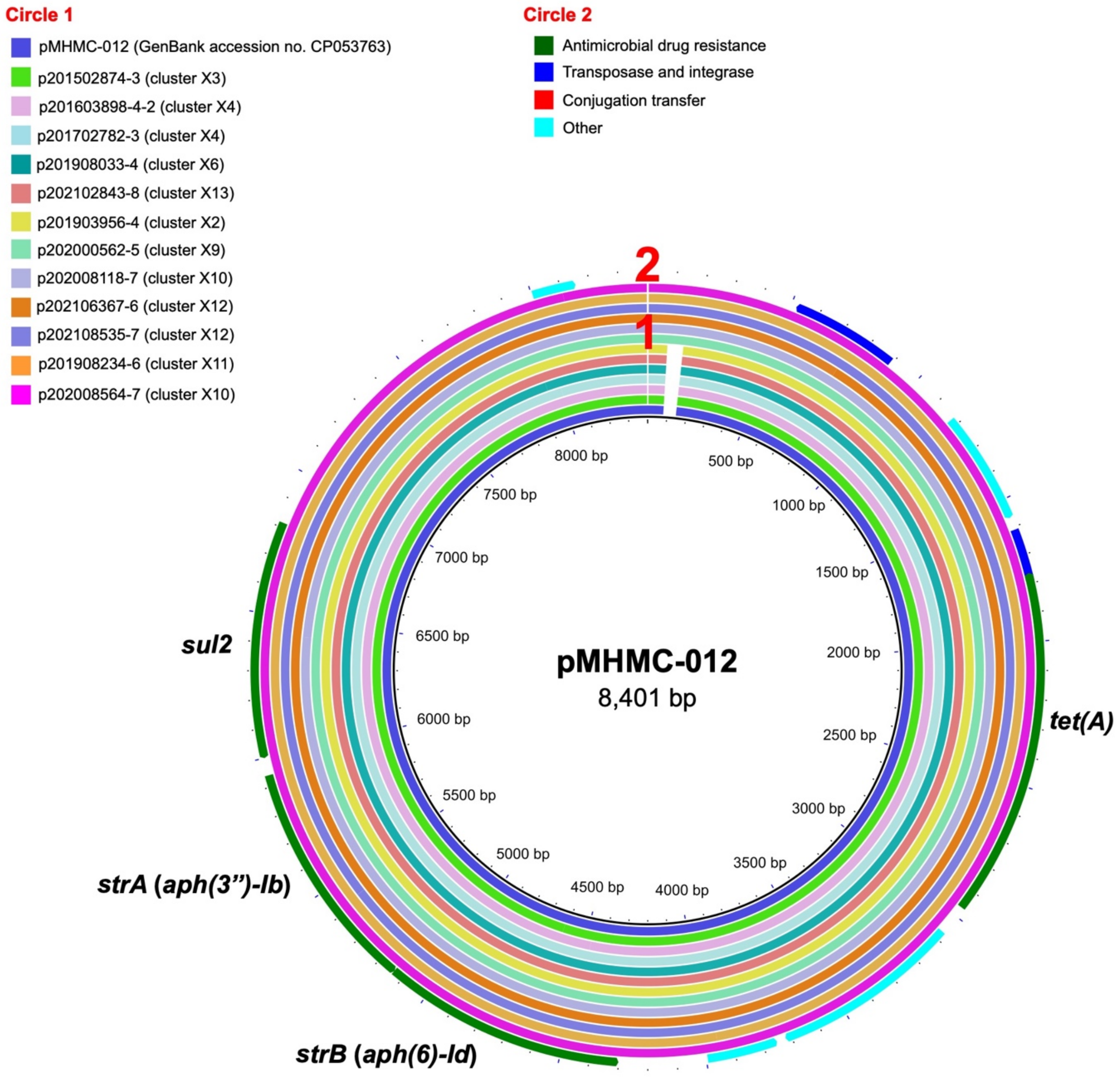

Circles from inside to outside indicate (1) the nucleotide position of pMHMC-012, a plasmid from a *S. sonnei* isolate acquired in Boston, USA in 2017 (ref. [1]), and regions of pMHMC-012 displaying high levels of identity to the sequences of 12 plasmids from XDR *S. sonnei* isolates in our study. The names of these plasmids and the XDR genomic clusters of their host *S. sonnei* isolates are shown in the legend, and (2) coding sequences (CDS) colored according to their functions. The antimicrobial drug resistance genes are indicated.

Supplementary Figure 2. Circular map and comparative analysis of IncF plasmids carrying the ESBL *bla*$_{CTX-M-27}$ gene

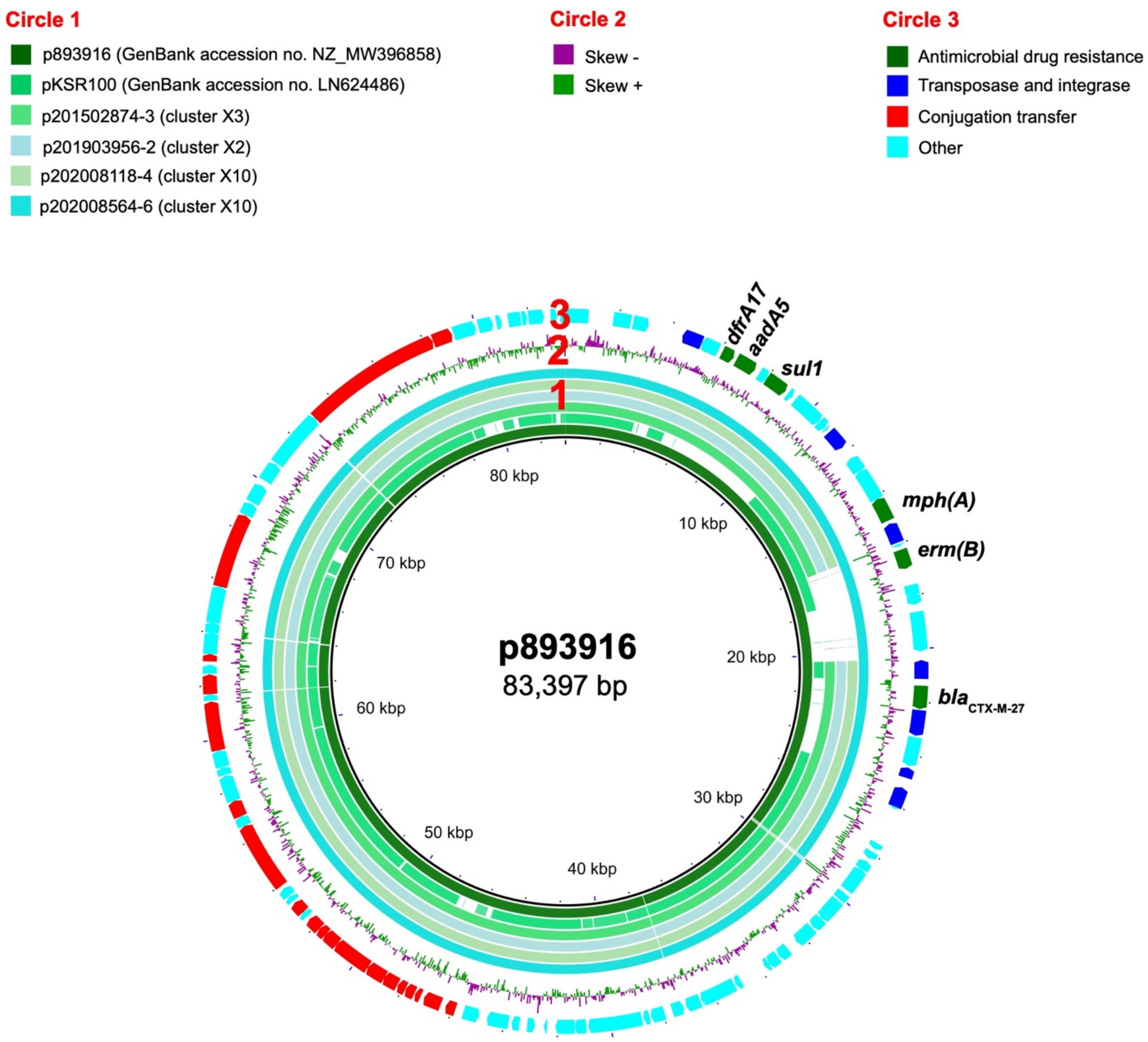

Circles from inside to outside indicate (1) the nucleotide position of p893916, a plasmid from a *S. sonnei* isolate collected in the UK in 2020 (ref. [2]), and regions of p893916 that displaying high levels of sequence identity to plasmids pKSR100 (from a *S. flexneri* 3a isolate, SF7955, collected in Canada in 2013)[3], p201502874-3 (XDR genomic cluster X3, our study), p201903956-2 (cluster X2, our study), p202008118-4 (cluster X10, our study), and p202008564-6 (cluster X10, our study), (2) a G+C content map of p893916, and (3) coding sequences (CDS) colored according to their functions. The antimicrobial drug resistance genes are indicated.

Supplementary Figure 3. Circular map and comparative analysis of IncI1 plasmids carrying the ESBL *bla*CTX-M-3 gene

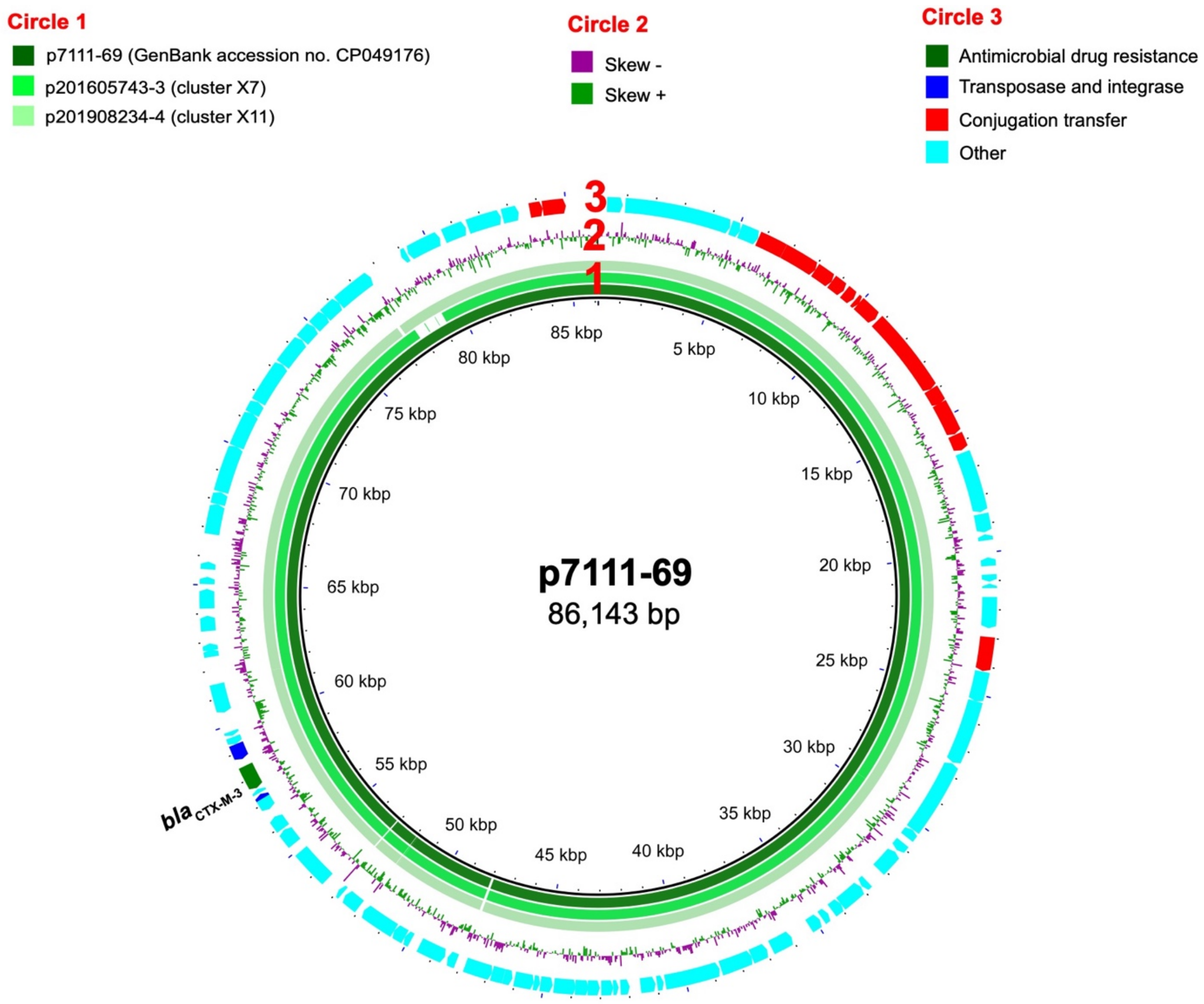

Circles from inside to outside indicate (1) the nucleotide position of p711-69, a plasmid from a *S. sonnei* isolate acquired in Turkey in 2019 (ref. [4]), and regions of p7111-69 displaying high levels of sequence identity to pKSR100 (from a *S. flexneri* 3a isolate, SF7955, collected in Canada in 2013)[3], p201605743-3 (XDR genomic cluster X7, our study), and p201908234-4 (cluster X11, our study), (2) a G+C content map of p711-69, and (3) coding sequences (CDS) colored according to their functions. The antimicrobial drug resistance gene is indicated.

Supplementary Figure 4. Circular map and comparative analysis of IncF plasmids carrying the ESBL *bla*$_{CTX-M-134}$ gene

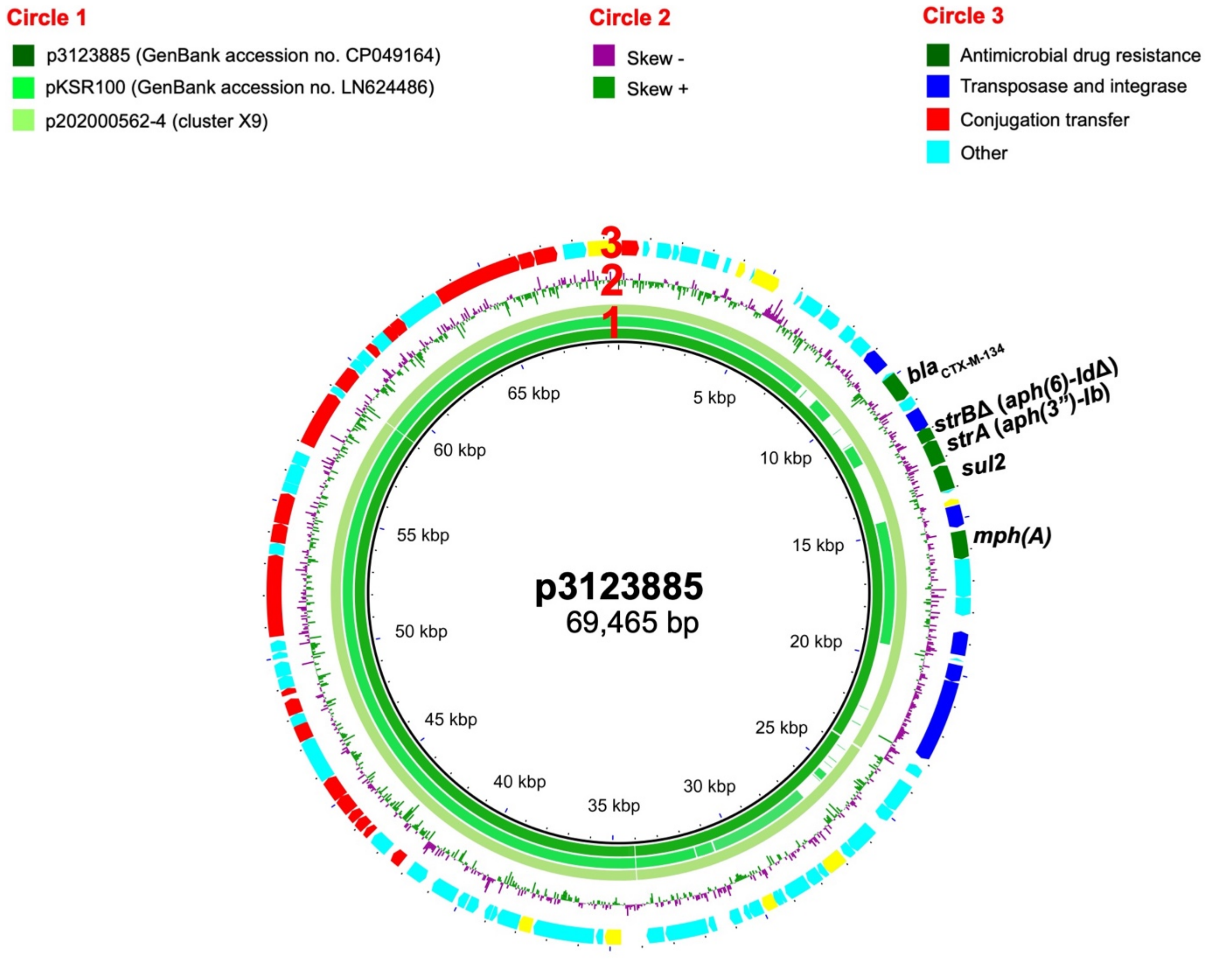

Circles from inside to outside indicate (1) the nucleotide position of p3123885, a plasmid from a *S. sonnei* isolate acquired in Israel in 2019 (ref. [4]), and regions of p3123885 displaying high levels of sequence identity to pKSR100 (from a *S. flexneri* 3a isolate, SF7955, collected in Canada in 2013)[3], and p202000562-4 (XDR genomic cluster X9, our study), (2) a G+C content map of p3123885, and (3) coding sequences (CDS) colored according to their functions. The antimicrobial drug resistance genes are indicated (most of the *strB* gene is deleted).

Supplementary Figure 5. Circular map and comparative analysis of IncF plasmids carrying the ESBL *bla*$_{CTX-M-15}$ gene

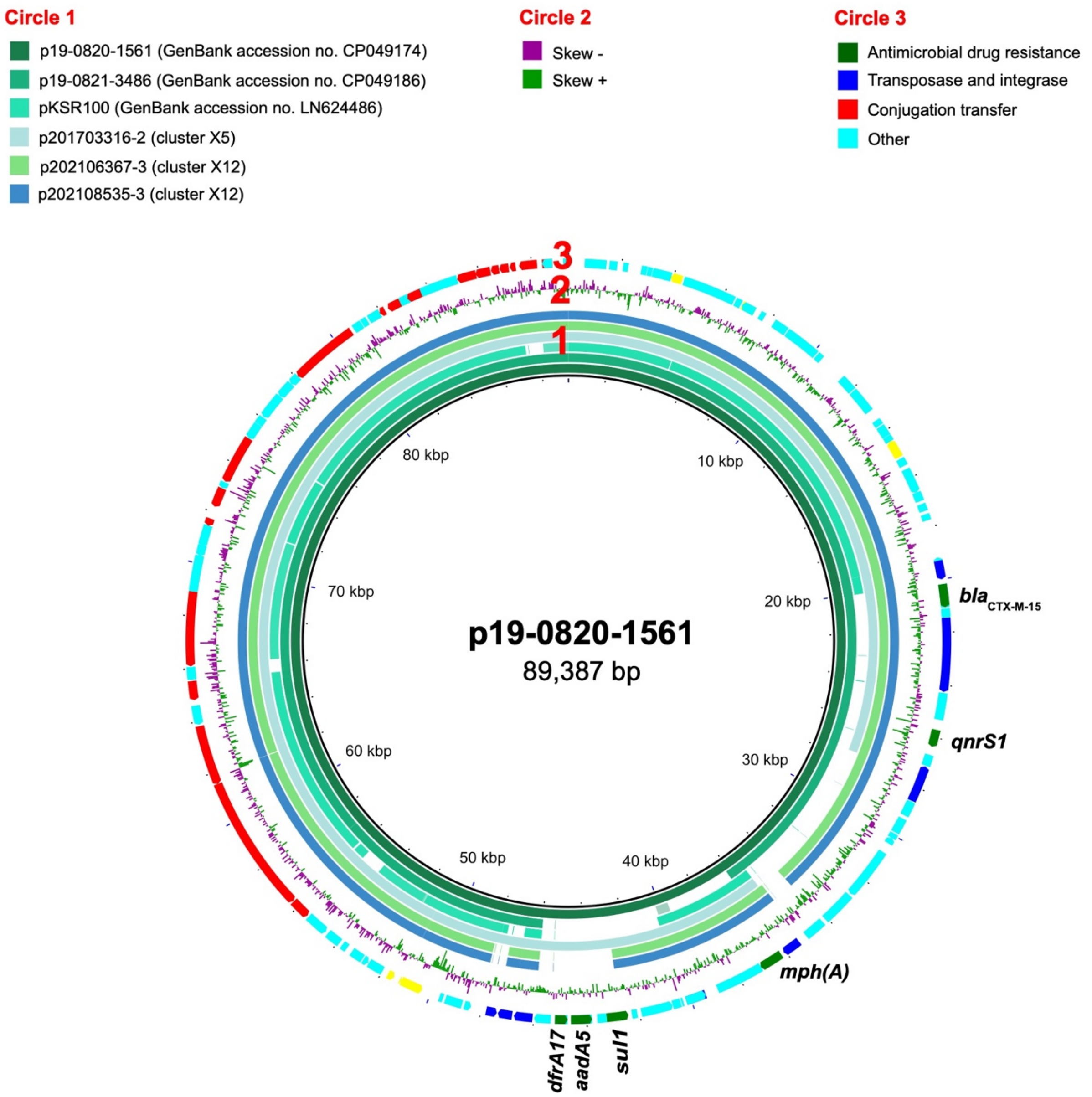

Circles from inside to outside indicate (1) the nucleotide position of p19-0820-1561, a plasmid from a *S. sonnei* isolate acquired in Nepal[4], and regions of p19-0820-1561 displaying high levels of sequence identity to plasmids p19-0821-3486 (from a *S. sonnei* isolate acquired in Egypt)[4], pKSR100 (from a *S. flexneri* 3a isolate, SF7955, collected in Canada, in 2013)[3], p201703316-2 (XDR genomic cluster X5, our study), p202106367-3 (cluster X12, our study), and p202108535-3 (cluster X12, our study), (2) a G+C content map of p19-0820-1561, and (3) coding sequences (CDS) colored according to their functions. The antimicrobial drug resistance genes are indicated.

Supplementary Figure 6. Representation of the genomic island containing the *bla*$_{CTX-M-15}$ gene in the XDR *S. sonnei* isolate 201908033

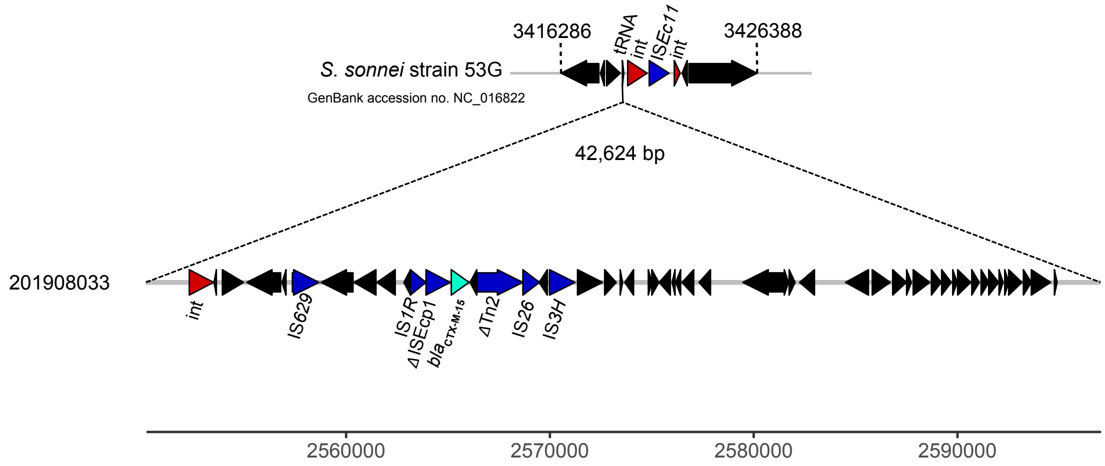

Supplementary Figure 7. Representation of the prophage containing the *bla*$_{CTX-M-15}$ gene in the XDR *S. sonnei* isolate 201701093

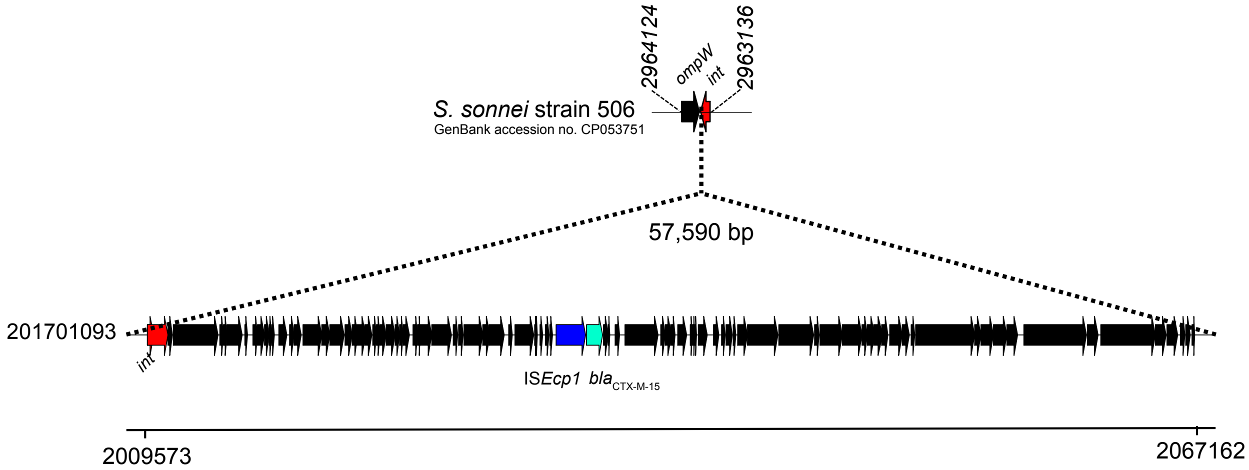

Supplementary Figure 8. Maximum-likelihood phylogeny of 232 *S. sonnei* genomic sequences from XDR (or inferred to be XDR) isolates.

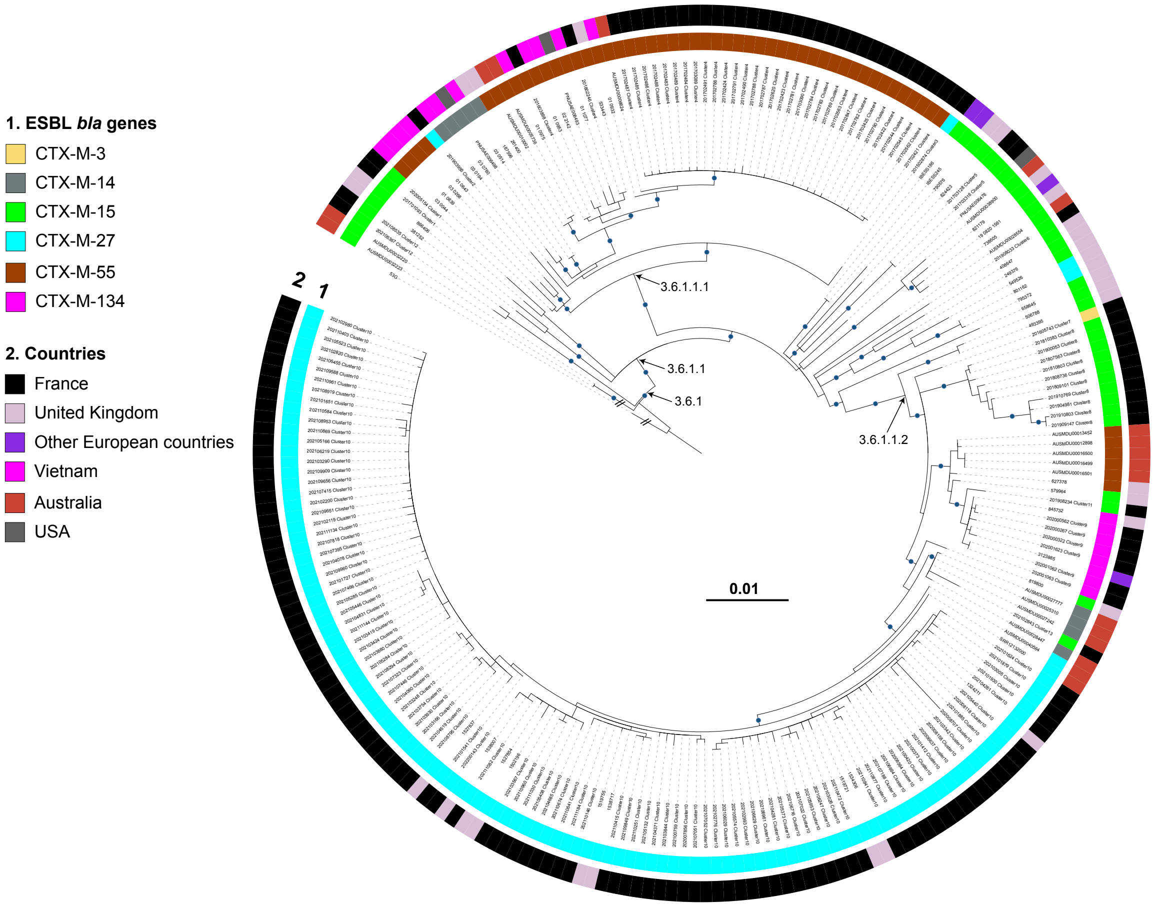

The dataset comprised the 164 French XDR, 67 international XDR (or inferred to be XDR) (Supplementary Table 4) *S. sonnei* isolates and the *S. sonnei* reference genome 53G. This reference genome (genotype 2.8.2) was used as the outgroup, and its branch length has been shortened by a factor of 100, as indicated by the double slash (//), to improve visualization. The length of the branch to genomes AUSMDU00032220 and AUSMDU00032223 (both belonging to genotype 3.6.3_Central Asia III) has been shortened by a factor of 10, also to improve visualization. Bootstrap support of more than 95% is indicated by blue circles. The strain name (and the XDR cluster for the French XDR isolates) is indicated at the tips of the branches. The rings show the associated information (see key) for each isolate, according to its position in the phylogeny, from the innermost to the outermost in the following order: (1) presence of *bla* genes encoding extended-spectrum beta-lactamases (ESBLs); (2) Countries of isolation of the isolates. The main *S. sonnei* genotypes are indicated. The scale bar indicates the number of substitutions per variable site (SNVs).

Supplementary Table 1. Phenotypic characteristics and metadata for the 164 XDR *S. sonnei* studied.

Isolate	Year	Age group (year)	Sex	French region	Travel	2017 school outbreak	Phenotypic antimicrobial drug resistance profile	MIC (mg/L)		
								CIP	AZM	CRO
201502874	2015	>15	M	Metropole	Unknown	No	AMP CRO STR SMX TMP SXT TET NAL CIP AZM	4	96	24
201603898	2016	>15	M	Metropole	Vietnam	No	AMP CRO STR GEN SMX TMP SXT TET NAL CIP AZM	2	48	32
201605743	2016	>15	M	Metropole	Unknown	No	AMP CRO STR SMX TMP SXT NAL CIP AZM	3	≥256	64
201701093	2017	>15	F	Metropole	None	No	AMP CRO SMX TMP SXT NAL CIP AZM	8	≥256	128
201702421	2017	0-15	F	Metropole	None	Yes	AMP CRO STR GEN SMX TMP SXT TET NAL CIP AZM	3	64	96
201702422	2017	0-15	F	Metropole	None	Yes	AMP CRO STR GEN SMX TMP SXT TET NAL CIP AZM	4	64	64
201702423	2017	0-15	M	Metropole	None	Yes	AMP CRO STR GEN SMX TMP SXT TET NAL CIP AZM	4	48	192
201702424	2017	0-15	F	Metropole	None	Yes	AMP CRO STR GEN SMX TMP SXT TET NAL CIP AZM	4	128	96
201702425	2017	0-15	M	Metropole	None	Yes	AMP CRO STR GEN SMX TMP SXT TET NAL CIP AZM	4	64	≥256
201702483	2017	0-15	F	Metropole	None	Yes	AMP CRO STR GEN SMX TMP SXT TET NAL CIP AZM	3	64	64
201702484	2017	0-15	M	Metropole	None	Yes	AMP CRO STR GEN SMX TMP SXT TET NAL CIP AZM	3	64	64
201702485	2017	0-15	M	Metropole	None	Yes	AMP CRO STR GEN SMX TMP SXT TET NAL CIP AZM	3	96	192
201702486	2017	0-15	M	Metropole	None	Yes	AMP CRO STR GEN SMX TMP SXT TET NAL CIP AZM	3	64	96
201702487	2017	0-15	M	Metropole	None	Yes	AMP CRO STR GEN SMX TMP SXT TET NAL CIP AZM	2	64	64
201702488	2017	0-15	F	Metropole	None	Yes	AMP CRO STR GEN SMX TMP SXT TET NAL CIP AZM	3	64	64
201702489	2017	0-15	M	Metropole	None	Yes	AMP CRO STR GEN SMX TMP SXT TET NAL CIP AZM	3	64	96
201702490	2017	0-15	F	Metropole	None	Yes	AMP CRO STR GEN SMX TMP SXT TET NAL CIP AZM	2	64	48
201702491	2017	0-15	M	Metropole	None	Yes	AMP CRO STR GEN SMX TMP SXT TET NAL CIP AZM	3	64	96
201702544	2017	0-15	F	Metropole	None	Yes	AMP CRO STR GEN SMX TMP SXT TET NAL CIP AZM	3	96	≥256
201702545	2017	0-15	M	Metropole	None	Yes	AMP CRO STR GEN SMX TMP SXT TET NAL CIP AZM	2	64	96
201702562	2017	0-15	M	Metropole	None	Yes	AMP CRO STR GEN SMX TMP SXT TET NAL CIP AZM	3	64	64
201702563	2017	0-15	F	Metropole	None	Yes	AMP CRO STR GEN SMX TMP SXT TET NAL CIP AZM	3	96	32
201702620	2017	>15	M	Metropole	None	Yes	AMP CRO STR GEN SMX TMP SXT TET NAL CIP AZM	3	128	≥256
201702781	2017	0-15	M	Metropole	Vietnam	Yes	AMP CRO STR GEN SMX TMP SXT TET NAL CIP AZM	3	96	≥256
201702782	2017	0-15	F	Metropole	None	Yes	AMP CRO STR GEN SMX TMP SXT TET NAL CIP AZM	4	128	64
201702783	2017	0-15	M	Metropole	None	Yes	AMP CRO STR GEN SMX TMP SXT TET NAL CIP AZM	3	96	96
201702784	2017	0-15	M	Metropole	None	Yes	AMP CRO STR GEN SMX TMP SXT TET NAL CIP AZM	3	96	64
201702786	2017	0-15	M	Metropole	None	Yes	AMP CRO STR GEN SMX TMP SXT TET NAL CIP AZM	3	96	192
201702787	2017	>15	M	Metropole	None	Yes	AMP CRO STR GEN SMX TMP SXT TET NAL CIP AZM	3	96	128
201702788	2017	>15	F	Metropole	None	Yes	AMP CRO STR GEN SMX TMP SXT TET NAL CIP AZM	3	48	128
201702789	2017	0-15	F	Metropole	None	Yes	AMP CRO STR GEN SMX TMP SXT TET NAL CIP AZM	3	128	96
201702790	2017	0-15	M	Metropole	None	Yes	AMP CRO STR GEN SMX TMP SXT TET NAL CIP AZM	3	96	96
201702791	2017	0-15	M	Metropole	None	Yes	AMP CRO STR GEN SMX TMP SXT TET NAL CIP AZM	3	128	64
201702847	2017	>15	F	Metropole	None	Yes	AMP CRO STR GEN SMX TMP SXT TET NAL CIP AZM	3	96	96
201703089	2017	>15	F	Metropole	None	Yes	AMP CRO STR GEN SMX TMP SXT TET NAL CIP AZM	>32	96	48
201703090	2017	0-15	M	Metropole	None	Yes	AMP CRO STR GEN SMX TMP SXT TET NAL CIP AZM	4	128	≥256
201703128	2017	0-15	F	Metropole	India	No	AMP CRO STR SMX TMP SXT NAL CIP AZM	6	128	12
201703316	2017	>15	F	Metropole	India	No	AMP CRO STR SMX TMP SXT NAL CIP AZM	6	192	16
201802246	2018	0-15	F	Metropole	Vietnam	No	AMP CRO STR SMX TMP SXT TET NAL CIP AZM	4	128	128
201807563	2018	>15	M	Metropole	None	No	AMP CRO STR SMX TMP SXT TET NAL CIP AZM	>32	≥256	128
201808736	2018	>15	M	Metropole	Unknown	No	AMP CRO STR SMX TMP SXT TET NAL CIP AZM	24	≥256	24
201809101	2018	>15	M	Metropole	None	No	AMP CRO STR SMX TMP SXT TET NAL CIP AZM	24	≥256	24

201810383	2018	>15	M	Metropole	None	No	AMP CRO STR SMX TMP SXT TET NAL CIP AZM	>32	≥256	12
201810803	2018	>15	M	Metropole	Spain	No	AMP CRO STR SMX TMP SXT TET NAL CIP AZM	24	≥256	32
201900053	2019	>15	M	Metropole	Unknown	No	AMP CRO STR SMX TMP SXT TET NAL CIP AZM	24	≥256	32
201903956	2019	>15	F	Metropole	Cambodia	No	AMP CRO STR SMX TMP SXT TET NAL CIP AZM	6	192	64
201904981	2019	>15	M	Metropole	None	No	AMP CRO STR SMX TMP SXT TET NAL CIP AZM	>32	≥256	24
201908033	2019	0-15	M	Metropole	None	No	AMP CRO STR SMX TMP SXT TET NAL CIP AZM	4	≥256	≥256
201908234	2019	>15	M	Metropole	None	No	AMP CRO STR SMX TMP SXT TET NAL CIP AZM	4	≥256	32
201909147	2019	>15	M	Metropole	None	No	AMP CRO STR SMX TMP SXT TET NAL CIP AZM	24	≥256	24
201910769	2019	>15	M	Metropole	Spain	No	AMP CRO STR SMX TMP SXT TET NAL CIP AZM	>32	≥256	128
201910803	2019	>15	M	Metropole	None	No	AMP CRO STR SMX TMP SXT TET NAL CIP AZM	>32	≥256	48
202000267	2020	>15	M	Metropole	None	No	AMP CRO STR SMX TMP SXT TET NAL CIP AZM	4	192	24
202000322	2020	>15	M	Metropole	Unknown	No	AMP CRO STR SMX TMP SXT TET NAL CIP AZM	4	192	48
202000562	2020	>15	M	Metropole	None	No	AMP CRO STR SMX TMP SXT TET NAL CIP AZM	3	192	48
202001062	2020	>15	M	Metropole	Switzerland	No	AMP CRO STR SMX TMP SXT TET NAL CIP AZM	4	192	96
202001063	2020	>15	F	Metropole	Spain	No	AMP CRO STR SMX TMP SXT TET NAL CIP AZM	4	192	96
202001623	2020	>15	M	Metropole	None	No	AMP CRO STR SMX TMP SXT TET NAL CIP AZM	4	192	128
202005154	2020	>15	F	La Réunion	None	No	AMP CRO SMX TMP SXT NAL CIP AZM	6	≥256	16
202006637	2020	>15	M	Metropole	None	No	AMP CRO STR SMX TMP SXT TET NAL CIP AZM	12	≥256	18
202007856	2020	>15	M	Metropole	None	No	AMP CRO STR SMX TMP SXT TET NAL CIP AZM	12	≥256	48
202008118	2020	>15	M	Metropole	None	No	AMP CRO STR SMX TMP SXT TET NAL CIP AZM	8	192	32
202008158	2020	>15	M	Metropole	None	No	AMP CRO STR SMX TMP SXT TET NAL CIP AZM	12	≥256	16
202008564	2020	>15	M	Metropole	None	No	AMP CRO STR SMX TMP SXT TET NAL CIP AZM	12	≥256	32
202008707	2020	>15	M	Metropole	None	No	AMP CRO STR SMX TMP SXT TET NAL CIP AZM	8	128	16
202100373	2021	>15	M	Metropole	Unknown	No	AMP CRO STR SMX TMP SXT TET NAL CIP AZM	8	192	24
202100420	2021	>15	M	Metropole	Unknown	No	AMP CRO STR SMX TMP SXT TET NAL CIP AZM	8	192	128
202100759	2021	>15	M	Metropole	Unknown	No	AMP CRO STR SMX TMP SXT TET NAL CIP AZM	12	≥256	16
202101412	2021	>15	M	Metropole	Unknown	No	AMP CRO STR SMX TMP SXT TET NAL CIP AZM	12	≥256	32
202101541	2021	>15	M	Metropole	None	No	AMP CRO STR SMX TMP SXT NAL CIP AZM	8	≥256	48
202101624	2021	>15	M	Metropole	Unknown	No	AMP CRO STR SMX TMP SXT TET NAL CIP AZM	12	≥256	96
202101651	2021	>15	M	Metropole	None	No	AMP CRO STR SMX TMP SXT TET NAL CIP AZM	32	≥256	64
202101727	2021	>15	M	Metropole	None	No	AMP CRO STR SMX TMP SXT TET NAL CIP AZM	12	≥256	24
202101879	2021	>15	M	Metropole	None	No	AMP CRO STR SMX TMP SXT TET NAL CIP AZM	6	≥256	32
202101885	2021	>15	M	Metropole	Unknown	No	AMP CRO STR SMX TMP SXT TET NAL CIP AZM	8	192	32
202101930	2021	>15	M	Metropole	Unknown	No	AMP CRO STR SMX TMP SXT TET NAL CIP AZM	12	≥256	24
202102119	2021	>15	M	Metropole	Unknown	No	AMP CRO STR SMX TMP SXT TET NAL CIP AZM	8	≥256	48
202102200	2021	>15	M	Metropole	Unknown	No	AMP CRO STR SMX TMP SXT TET NAL CIP AZM	8	≥256	32
202102776	2021	>15	M	Metropole	None	No	AMP CRO STR SMX TMP SXT TET NAL CIP AZM	8	≥256	48
202102820	2021	>15	M	Metropole	None	No	AMP CRO STR SMX TMP SXT TET NAL CIP AZM	8	≥256	24
202102843	2021	>15	M	Metropole	None	No	AMP CRO STR SMX TMP SXT TET NAL CIP AZM	6	≥256	12
202102980	2021	>15	M	Metropole	None	No	AMP CRO STR SMX TMP SXT TET NAL CIP AZM	6	128	24
202102993	2021	>15	M	Metropole	Unknown	No	AMP CRO STR SMX TMP SXT TET NAL CIP AZM	8	≥256	64
202103005	2021	>15	M	Metropole	None	No	AMP CRO STR SMX TMP SXT TET NAL CIP AZM	6	96	24
202103026	2021	>15	M	Metropole	None	No	AMP CRO STR SMX TMP SXT TET NAL CIP AZM	8	≥256	32
202103166	2021	>15	M	Metropole	None	No	AMP CRO STR SMX TMP SXT TET NAL CIP AZM	6	≥256	24
202103248	2021	>15	M	Metropole	None	No	AMP CRO STR SMX TMP SXT TET NAL CIP AZM	6	≥256	24
202103290	2021	>15	M	Metropole	None	No	AMP CRO STR SMX TMP SXT TET NAL CIP AZM	6	32	24
202103342	2021	>15	M	Metropole	None	No	AMP CRO STR SMX TMP SXT TET NAL CIP AZM	6	96	24
202103419	2021	>15	M	Metropole	None	No	AMP CRO STR SMX TMP SXT TET NAL CIP AZM	6	96	24
202103424	2021	>15	M	Metropole	Cameroon	No	AMP CRO STR SMX TMP SXT TET NAL CIP AZM	6	32	48
202103567	2021	>15	M	Metropole	Unknown	No	AMP CRO STR SMX TMP SXT TET NAL CIP AZM	8	≥256	24
202103754	2021	>15	M	Metropole	Unknown	No	AMP CRO STR SMX TMP SXT TET NAL CIP AZM	6	≥256	32

202103844	2021	>15	M	Metropole	Unknown	No	AMP CRO STR SMX TMP SXT TET NAL CIP AZM	6	≥256	48
202103880	2021	>15	M	Metropole	None	No	AMP CRO STR SMX TMP SXT TET NAL CIP AZM	8	48	32
202103930	2021	>15	M	Metropole	None	No	AMP CRO STR SMX TMP SXT TET NAL CIP AZM	6	≥256	32
202104060	2021	>15	F	Metropole	Unknown	No	AMP CRO STR SMX TMP SXT TET NAL CIP AZM	4	≥256	32
202104078	2021	>15	M	Metropole	None	No	AMP CRO STR SMX TMP SXT TET NAL CIP AZM	8	128	48
202104261	2021	>15	M	Metropole	None	No	AMP CRO STR SMX TMP SXT TET NAL CIP AZM	6	≥256	16
202104281	2021	>15	M	Metropole	None	No	AMP CRO STR SMX TMP SXT TET NAL CIP AZM	24	≥256	96
202104371	2021	>15	M	Metropole	Unknown	No	AMP CRO STR SMX TMP SXT TET NAL CIP AZM	6	≥256	24
202104519	2021	>15	M	Metropole	None	No	AMP CRO STR SMX TMP SXT TET NAL CIP AZM	6	≥256	16
202104685	2021	>15	M	Metropole	Unknown	No	AMP CRO STR SMX TMP SXT TET NAL CIP AZM	6	≥256	16
202104831	2021	>15	M	Metropole	None	No	AMP CRO STR SMX TMP SXT TET NAL CIP AZM	8	≥256	48
202105132	2021	>15	M	Metropole	Unknown	No	AMP CRO STR SMX TMP SXT TET NAL CIP AZM	8	192	32
202105166	2021	>15	M	Metropole	Unknown	No	AMP CRO STR SMX TMP SXT TET NAL CIP AZM	6	≥256	32
202105284	2021	>15	M	Metropole	None	No	AMP CRO STR SMX TMP SXT TET NAL CIP AZM	8	≥256	192
202105285	2021	>15	M	Metropole	None	No	AMP CRO STR SMX TMP SXT TET NAL CIP AZM	12	96	48
202105373	2021	>15	M	Metropole	Unknown	No	AMP CRO STR SMX TMP SXT TET NAL CIP AZM	16	≥256	24
202105408	2021	>15	M	Metropole	Unknown	No	AMP CRO STR SMX TMP SXT TET NAL CIP AZM	12	≥256	24
202105440	2021	>15	M	Metropole	Unknown	No	AMP CRO STR SMX TMP SXT TET NAL CIP AZM	8	≥256	192
202105446	2021	>15	M	Metropole	Unknown	No	AMP CRO TMP NAL CIP AZM	12	48	48
202105523	2021	>15	M	Metropole	None	No	AMP CRO STR SMX TMP SXT TET NAL CIP AZM	4	≥256	64
202105574	2021	>15	M	Metropole	None	No	AMP CRO STR SMX TMP SXT TET NAL CIP AZM	12	≥256	64
202105953	2021	>15	F	Metropole	Unknown	No	AMP CRO STR SMX TMP SXT TET NAL CIP AZM	12	≥256	32
202106029	2021	>15	M	Metropole	Unknown	No	AMP CRO STR SMX TMP SXT TET NAL CIP AZM	8	≥256	32
202106219	2021	>15	M	Metropole	None	No	AMP CRO STR SMX TMP SXT TET NAL CIP AZM	8	≥256	24
202106247	2021	0-15	M	Metropole	Unknown	No	AMP CRO STR SMX TMP SXT TET NAL CIP AZM	12	≥256	16
202106367	2021	>15	F	Metropole	Lebanon	No	AMP CRO STR SMX TMP SXT TET NAL CIP AZM	1	96	6
202106455	2021	>15	M	Metropole	None	No	AMP CRO STR SMX TMP SXT TET NAL CIP AZM	4	≥256	24
202106629	2021	>15	M	Metropole	Unknown	No	AMP CRO STR SMX TMP SXT TET NAL CIP AZM	32	≥256	24
202106664	2021	>15	M	Metropole	None	No	AMP CRO STR SMX TMP SXT NAL CIP AZM	16	≥256	24
202106716	2021	>15	M	Metropole	Spain	No	AMP CRO STR SMX TMP SXT TET NAL CIP AZM	12	≥256	24
202106961	2021	>15	M	Metropole	Unknown	No	AMP CRO STR SMX TMP SXT TET NAL CIP AZM	32	≥256	12
202107022	2021	>15	M	Metropole	None	No	AMP CRO STR SMX TMP SXT TET NAL CIP AZM	12	≥256	32
202107051	2021	>15	M	Metropole	None	No	AMP CRO STR SMX TMP SXT TET NAL CIP AZM	16	≥256	48
202107052	2021	>15	M	Metropole	None	No	AMP CRO STR SMX TMP SXT TET NAL CIP AZM	16	≥256	32
202107188	2021	>15	M	Metropole	Unknown	No	AMP CRO STR SMX TMP SXT NAL CIP AZM	16	≥256	32
202107323	2021	>15	M	Metropole	Unknown	No	AMP CRO STR SMX TMP SXT TET NAL CIP AZM	16	≥256	32
202107395	2021	>15	M	Metropole	Unknown	No	AMP CRO STR SMX TMP SXT TET NAL CIP AZM	6	≥256	48
202107415	2021	>15	M	Metropole	Unknown	No	AMP CRO STR SMX TMP SXT TET NAL CIP AZM	6	≥256	48
202107446	2021	>15	M	Metropole	None	No	AMP CRO STR SMX TMP SXT TET NAL CIP AZM	12	≥256	48
202107466	2021	>15	M	Metropole	None	No	AMP CRO STR SMX TMP SXT TET NAL CIP AZM	6	≥256	192
202107818	2021	>15	M	Metropole	Unknown	No	AMP CRO STR SMX TMP SXT TET NAL CIP AZM	6	≥256	32
202108294	2021	>15	M	Metropole	Unknown	No	AMP CRO STR SMX TMP SXT TET NAL CIP AZM	12	≥256	24
202108535	2021	>15	F	Metropole	None	No	AMP CRO STR SMX TMP SXT TET NAL CIP AZM	1	96	16
202108795	2021	>15	M	Metropole	Unknown	No	AMP CRO STR SMX TMP SXT TET NAL CIP AZM	6	≥256	24
202108919	2021	>15	M	Metropole	None	No	AMP CRO STR SMX TMP SXT TET NAL CIP AZM	4	≥256	24
202108953	2021	>15	M	Metropole	Unknown	No	AMP CRO STR SMX TMP SXT TET NAL CIP AZM	8	≥256	48
202109588	2021	>15	M	Metropole	Unknown	No	AMP CRO STR SMX TMP SXT TET NAL CIP AZM	6	≥256	24
202109651	2021	>15	M	Metropole	None	No	AMP CRO STR SMX TMP SXT TET NAL CIP AZM	4	≥256	24
202109656	2021	>15	M	Metropole	Unknown	No	AMP CRO STR SMX TMP SXT TET NAL CIP AZM	16	≥256	24
202109849	2021	>15	M	Metropole	Unknown	No	AMP CRO STR SMX TMP SXT NAL CIP AZM	12	≥256	24
202109909	2021	>15	M	Metropole	Unknown	No	AMP CRO STR SMX TMP SXT TET NAL CIP AZM	4	≥256	96

202109960	2021	>15	M	Metropole	Unknown	No	AMP CRO STR SMX TMP SXT TET NAL CIP AZM	6	≥256	48
202110146	2021	>15	M	Metropole	Unknown	No	AMP CRO STR SMX TMP SXT NAL CIP AZM	8	≥256	32
202110251	2021	>15	M	Metropole	Unknown	No	AMP CRO STR SMX TMP SXT NAL CIP AZM	8	≥256	24
202110403	2021	>15	M	Metropole	Unknown	No	AMP CRO STR SMX TMP SXT TET NAL CIP AZM	12	≥256	32
202110415	2021	>15	M	Metropole	None	No	AMP CRO STR SMX TMP SXT NAL CIP AZM	8	≥256	32
202110472	2021	>15	M	Metropole	None	No	AMP CRO STR SMX TMP SXT TET NAL CIP AZM	8	≥256	32
202110584	2021	>15	M	Metropole	Unknown	No	AMP CRO STR SMX TMP SXT NAL CIP AZM	8	≥256	32
202110641	2021	>15	M	Metropole	Unknown	No	AMP CRO STR SMX TMP SXT NAL CIP AZM	8	≥256	32
202110674	2021	>15	M	Metropole	Unknown	No	AMP CRO STR SMX TMP SXT NAL CIP AZM	8	≥256	24
202110841	2021	>15	M	Metropole	Spain	No	AMP CRO STR SMX TMP SXT NAL CIP AZM	8	≥256	16
202110869	2021	>15	F	Metropole	Unknown	No	AMP CRO STR SMX TMP SXT NAL CIP AZM	16	≥256	48
202110877	2021	>15	M	Metropole	Unknown	No	AMP CRO STR SMX TMP SXT NAL CIP AZM	12	≥256	48
202110960	2021	>15	M	Metropole	Unknown	No	AMP CRO STR SMX TMP SXT TET NAL CIP AZM	16	≥256	24
202110961	2021	>15	M	Metropole	Unknown	No	AMP CRO STR SMX TMP SXT TET NAL CIP AZM	12	≥256	32
202111030	2021	>15	M	Metropole	Unknown	No	AMP CRO STR SMX TMP SXT TET NAL CIP AZM	12	≥256	24
202111082	2021	>15	M	Metropole	Unknown	No	AMP CRO STR SMX TMP SXT TET NAL CIP AZM	12	≥256	32
202111134	2021	>15	M	Metropole	Unknown	No	AMP CRO STR SMX TMP SXT TET NAL CIP AZM	4	≥256	24
202111144	2021	>15	M	Metropole	Unknown	No	AMP CRO STR SMX TMP SXT TET NAL CIP AZM	8	192	48
202111184	2021	>15	M	Metropole	None	No	AMP CRO STR SMX TMP SXT NAL CIP AZM	12	≥256	24
202200143	2021	>15	M	Metropole	Unknown	No	AMP CRO STR SMX TMP SXT NAL CIP AZM	8	≥256	48

M, male; F, female; MIC; minimum inhibitory concentration; AMP, ampicillin; CRO, ceftriaxone; STR, streptomycin; SMX, sulfamethoxazole; TMP, trimethoprim; TET, tetracycline; NAL, nalidixic acid; CIP, ciprofloxacin; AZM, azithromycin.

Supplementary Table 2. Genomic characteristics for the 164 XDR *S. sonnei* isolates under study.

Isolate	Cluster	Genotype	Genotype alias	Acquired antimicrobial drug resistance genes	QRDR mutation *gyrA*	QRDR mutation *parC*	Accession no.
201701093	X1	3.6.1.1	CipR	*bla*$_{\text{CTX-M-15}}$, *sul1, dfrA1, dfrA5, qnrS13, mph(A), erm(B)*	S83L	S80I	ERS6492764
202005154	X1	3.6.1.1	CipR	*bla*$_{\text{CTX-M-15}}$, *sul1, dfrA1, dfrA5, qnrS13, mph(A), erm(B)*	S83L	S80I	ERS6576287
201903956	X2	3.6.1.1.1	CipR.SEA	*bla*$_{\text{CTX-M-27}}$, *strA, strB, aadA5, sul1, sul2, dfrA1, dfrA17, tet(A), mph(A)*	S83L, D87G	S80I	ERS6495067
201502874	X3	3.6.1.1.1	CipR.SEA	*bla*$_{\text{CTX-M-27}}$, *strA, strB, aadA5, sul1, sul2, dfrA1, dfrA17, tet(A), mph(A)*	S83L, D87G	S80I	ERS12446082
201603898	X4	3.6.1.1.1	CipR.SEA	*bla*$_{\text{CTX-M-55}}$, *strA, strB, aac(3)-IIa, sul2, dfrA1, tet(A), mph(A)*	S83L, D87G	S80I	ERS12446174
201702421	X4	3.6.1.1.1	CipR.SEA	*bla*$_{\text{CTX-M-55}}$, *strA, strB, aac(3)-IIa, sul2, dfrA1, tet(A), mph(A)*	S83L, D87G	S80I	ERS6495734
201702422	X4	3.6.1.1.1	CipR.SEA	*bla*$_{\text{CTX-M-55}}$, *strA, strB, aac(3)-IIa, sul2, dfrA1, tet(A), mph(A)*	S83L, D87G	S80I	ERS6495735
201702423	X4	3.6.1.1.1	CipR.SEA	*bla*$_{\text{CTX-M-55}}$, *strA, strB, aac(3)-IIa, sul2, dfrA1, tet(A), mph(A)*	S83L, D87G	S80I	ERS6495736
201702424	X4	3.6.1.1.1	CipR.SEA	*bla*$_{\text{CTX-M-55}}$, *strA, strB, aac(3)-IIa, sul2, dfrA1, tet(A), mph(A)*	S83L, D87G	S80I	ERS6495737
201702425	X4	3.6.1.1.1	CipR.SEA	*bla*$_{\text{CTX-M-55}}$, *strA, strB, aac(3)-IIa, sul2, dfrA1, tet(A), mph(A)*	S83L, D87G	S80I	ERS6495738
201702483	X4	3.6.1.1.1	CipR.SEA	*bla*$_{\text{CTX-M-55}}$, *strA, strB, aac(3)-IIa, sul2, dfrA1, tet(A), mph(A)*	S83L, D87G	S80I	ERS6495742
201702484	X4	3.6.1.1.1	CipR.SEA	*bla*$_{\text{CTX-M-55}}$, *strA, strB, aac(3)-IIa, sul2, dfrA1, tet(A), mph(A)*	S83L, D87G	S80I	ERS6495743
201702485	X4	3.6.1.1.1	CipR.SEA	*bla*$_{\text{CTX-M-55}}$, *strA, strB, aac(3)-IIa, sul2, dfrA1, tet(A), mph(A)*	S83L, D87G	S80I	ERS6495744
201702486	X4	3.6.1.1.1	CipR.SEA	*bla*$_{\text{CTX-M-55}}$, *strA, strB, aac(3)-IIa, sul2, dfrA1, tet(A), mph(A)*	S83L, D87G	S80I	ERS6495745
201702487	X4	3.6.1.1.1	CipR.SEA	*bla*$_{\text{CTX-M-55}}$, *strA, strB, aac(3)-IIa, sul2, dfrA1, tet(A), mph(A)*	S83L, D87G	S80I	ERS6495746
201702488	X4	3.6.1.1.1	CipR.SEA	*bla*$_{\text{CTX-M-55}}$, *strA, strB, aac(3)-IIa, sul2, dfrA1, tet(A), mph(A)*	S83L, D87G	S80I	ERS6495747
201702489	X4	3.6.1.1.1	CipR.SEA	*bla*$_{\text{CTX-M-55}}$, *strA, strB, aac(3)-IIa, sul2, dfrA1, tet(A), mph(A)*	S83L, D87G	S80I	ERS6495748
201702490	X4	3.6.1.1.1	CipR.SEA	*bla*$_{\text{CTX-M-55}}$, *strA, strB, aac(3)-IIa, sul2, dfrA1, tet(A), mph(A)*	S83L, D87G	S80I	ERS6495749
201702491	X4	3.6.1.1.1	CipR.SEA	*bla*$_{\text{CTX-M-55}}$, *strA, strB, aac(3)-IIa, sul2, dfrA1, tet(A), mph(A)*	S83L, D87G	S80I	ERS6495750
201702544	X4	3.6.1.1.1	CipR.SEA	*bla*$_{\text{CTX-M-55}}$, *strA, strB, aac(3)-IIa, sul2, dfrA1, tet(A), mph(A)*	S83L, D87G	S80I	ERS6495756
201702545	X4	3.6.1.1.1	CipR.SEA	*bla*$_{\text{CTX-M-55}}$, *strA, strB, aac(3)-IIa, sul2, dfrA1, tet(A), mph(A)*	S83L, D87G	S80I	ERS6495757
201702562	X4	3.6.1.1.1	CipR.SEA	*bla*$_{\text{CTX-M-55}}$, *strA, strB, aac(3)-IIa, sul2, dfrA1, tet(A), mph(A)*	S83L, D87G	S80I	ERS6495758
201702563	X4	3.6.1.1.1	CipR.SEA	*bla*$_{\text{CTX-M-55}}$, *strA, strB, aac(3)-IIa, sul2, dfrA1, tet(A), mph(A)*	S83L, D87G	S80I	ERS6495759
201702620	X4	3.6.1.1.1	CipR.SEA	*bla*$_{\text{CTX-M-55}}$, *strA, strB, aac(3)-IIa, sul2, dfrA1, tet(A), mph(A)*	S83L, D87G	S80I	ERS6495761
201702781	X4	3.6.1.1.1	CipR.SEA	*bla*$_{\text{CTX-M-55}}$, *strA, strB, aac(3)-IIa, sul2, dfrA1, tet(A), mph(A)*	S83L, D87G	S80I	ERS6495763
201702782	X4	3.6.1.1.1	CipR.SEA	*bla*$_{\text{CTX-M-55}}$, *strA, strB, aac(3)-IIa, sul2, dfrA1, tet(A), mph(A)*	S83L, D87G	S80I	ERS6495764
201702783	X4	3.6.1.1.1	CipR.SEA	*bla*$_{\text{CTX-M-55}}$, *strA, strB, aac(3)-IIa, sul2, dfrA1, tet(A), mph(A)*	S83L, D87G	S80I	ERS6495765
201702784	X4	3.6.1.1.1	CipR.SEA	*bla*$_{\text{CTX-M-55}}$, *strA, strB, aac(3)-IIa, sul2, dfrA1, tet(A), mph(A)*	S83L, D87G	S80I	ERS6495766
201702786	X4	3.6.1.1.1	CipR.SEA	*bla*$_{\text{CTX-M-55}}$, *strA, strB, aac(3)-IIa, sul2, dfrA1, tet(A), mph(A)*	S83L, D87G	S80I	ERS6495768
201702787	X4	3.6.1.1.1	CipR.SEA	*bla*$_{\text{CTX-M-55}}$, *strA, strB, aac(3)-IIa, sul2, dfrA1, tet(A), mph(A)*	S83L, D87G	S80I	ERS6575823
201702788	X4	3.6.1.1.1	CipR.SEA	*bla*$_{\text{CTX-M-55}}$, *strA, strB, aac(3)-IIa, sul2, dfrA1, tet(A), mph(A)*	S83L, D87G	S80I	ERS6575824
201702789	X4	3.6.1.1.1	CipR.SEA	*bla*$_{\text{CTX-M-55}}$, *strA, strB, aac(3)-IIa, sul2, dfrA1, tet(A), mph(A)*	S83L, D87G	S80I	ERS6575825
201702790	X4	3.6.1.1.1	CipR.SEA	*bla*$_{\text{CTX-M-55}}$, *strA, strB, aac(3)-IIa, sul2, dfrA1, tet(A), mph(A)*	S83L, D87G	S80I	ERS6575826
201702791	X4	3.6.1.1.1	CipR.SEA	*bla*$_{\text{CTX-M-55}}$, *strA, strB, aac(3)-IIa, sul2, dfrA1, tet(A), mph(A)*	S83L, D87G	S80I	ERS6575827
201702847	X4	3.6.1.1.1	CipR.SEA	*bla*$_{\text{CTX-M-55}}$, *strA, strB, aac(3)-IIa, sul2, dfrA1, tet(A), mph(A)*	S83L, D87G	S80I	ERS6575830
201703089	X4	3.6.1.1.1	CipR.SEA	*bla*$_{\text{CTX-M-55}}$, *strA, strB, aac(3)-IIa, sul2, dfrA1, tet(A), mph(A)*	S83L, D87G	S80I	ERS6575842
201703090	X4	3.6.1.1.1	CipR.SEA	*bla*$_{\text{CTX-M-55}}$, *strA, strB, aac(3)-IIa, sul2, dfrA1, tet(A), mph(A)*	S83L, D87G	S80I	ERS6575843

201802246	X4	3.6.1.1.1	CipR.SEA	*bla*$_{CTX-M-55}$, *strA, strB, sul2, dfrA1, tet(A), mph(A)*	S83L, D87G	S80I	ERS6575238
201703128	X5	3.6.1.1	CipR	*bla*$_{CTX-M-15}$, *aadA5, sul1, dfrA1, dfrA17, qnrS1, mph(A)*	S83L, D87G	S80I	ERS6575844
201703316	X5	3.6.1.1	CipR	*bla*$_{CTX-M-15}$, *aadA5, sul1, dfrA1, dfrA17, qnrS1, mph(A)*	S83L, D87G	S80I	ERS6575847
201908033	X6	3.6.1.1	CipR	*bla*$_{CTX-M-15}$, *strA, strB, sul2, dfrA1, tet(A), mph(A), erm(B)*	S83L, D87G	S80I	ERS6495503
201605743	X7	3.6.1.1.2	CipR.MSM5	*bla*$_{TEM-1B}$, *bla*$_{CTX-M-3}$, *aadA5, sul1, dfrA1, dfrA17, mph(A), erm(B)*	S83L, D87G	S80I	ERS12446187
201807563	X8	3.6.1.1.2	CipR.MSM5	*bla*$_{TEM-1B}$, *bla*$_{CTX-M-15}$, *aadA1, aadA5, sul1, sul2, dfrA1, dfrA17, tet(B), qnrS1, mph(A), erm(B)*	S83L, D87G	S80I	ERS6575531
201808736	X8	3.6.1.1.2	CipR.MSM5	*bla*$_{TEM-1B}$, *bla*$_{CTX-M-15}$, *aadA1, aadA5, sul1, sul2, dfrA1, dfrA17, tet(B), qnrS1, mph(A), erm(B)*	S83L, D87G	S80I	ERS6492807
201809101	X8	3.6.1.1.2	CipR.MSM5	*bla*$_{TEM-1B}$, *bla*$_{CTX-M-15}$, *aadA1, aadA5, sul1, sul2, dfrA1, dfrA17, tet(B), qnrS1, mph(A), erm(B)*	S83L, D87G	S80I	ERS6492833
201810383	X8	3.6.1.1.2	CipR.MSM5	*bla*$_{TEM-1B}$, *bla*$_{CTX-M-15}$, *aadA1, aadA5, sul1, sul2, dfrA1, dfrA17, tet(B), qnrS1, mph(A), erm(B)*	S83L, D87G	S80I	ERS6494748
201810803	X8	3.6.1.1.2	CipR.MSM5	*bla*$_{TEM-1B}$, *bla*$_{CTX-M-15}$, *aadA1, aadA5, sul1, sul2, dfrA1, dfrA17, tet(B), qnrS1, mph(A), erm(B)*	S83L, D87G	S80I	ERS6494788
201900053	X8	3.6.1.1.2	CipR.MSM5	*bla*$_{TEM-1B}$, *bla*$_{CTX-M-15}$, *aadA1, aadA5, sul1, sul2, dfrA1, dfrA17, tet(B), qnrS1, mph(A), erm(B)*	S83L, D87G	S80I	ERS6575939
201904981	X8	3.6.1.1.2	CipR.MSM5	*bla*$_{TEM-1B}$, *bla*$_{CTX-M-15}$, *aadA1, aadA5, sul1, sul2, dfrA1, dfrA17, tet(B), qnrS1, mph(A), erm(B)*	S83L, D87G	S80I	ERS6495091
201909147	X8	3.6.1.1.2	CipR.MSM5	*bla*$_{TEM-1B}$, *bla*$_{CTX-M-15}$, *aadA1, aadA5, sul1, sul2, dfrA1, dfrA17, tet(B), qnrS1, mph(A), erm(B)*	S83L, D87G	S80I	ERS6495579
201910769	X8	3.6.1.1.2	CipR.MSM5	*bla*$_{TEM-1B}$, *bla*$_{CTX-M-15}$, *aadA1, aadA5, sul1, sul2, dfrA1, dfrA17, tet(B), qnrS1, mph(A), erm(B)*	S83L, D87G	S80I	ERS6575039
201910803	X8	3.6.1.1.2	CipR.MSM5	*bla*$_{TEM-1B}$, *bla*$_{CTX-M-15}$, *aadA1, aadA5, sul1, sul2, dfrA1, dfrA17, tet(B), qnrS1, mph(A), erm(B)*	S83L, D87G	S80I	ERS6575045
202000267	X9	3.6.1.1.2	CipR.MSM5	*bla*$_{CTX-M-134}$, *strA, strB, sul2, dfrA1, tet(A), mph(A)*	S83L, D87G	S80I	ERS6495836
202000322	X9	3.6.1.1.2	CipR.MSM5	*bla*$_{CTX-M-134}$, *strA, strB, sul2, dfrA1, tet(A), mph(A)*	S83L, D87G	S80I	ERS6495841
202000562	X9	3.6.1.1.2	CipR.MSM5	*bla*$_{CTX-M-134}$, *strA, strB, sul2, dfrA1, tet(A), mph(A)*	S83L, D87G	S80I	ERS6575389
202001062	X9	3.6.1.1.2	CipR.MSM5	*bla*$_{CTX-M-134}$, *strA, strB, sul2, dfrA1, tet(A), mph(A)*	S83L, D87G	S80I	ERS6576215
202001063	X9	3.6.1.1.2	CipR.MSM5	*bla*$_{CTX-M-134}$, *strA, strB, sul2, dfrA1, tet(A), mph(A)*	S83L, D87G	S80I	ERS6576216
202001623	X9	3.6.1.1.2	CipR.MSM5	*bla*$_{CTX-M-134}$, *strA, strB, sul2, dfrA1, tet(A), mph(A)*	S83L, D87G	S80I	ERS6576007
202006637	X10	3.6.1.1.2	CipR.MSM5	*bla*$_{CTX-M-27}$, *strA, strB, aadA5, sul1, sul2, dfrA1, dfrA17, tet(A), qnrB19, mph(A), erm(B)*	S83L, D87G	S80I	ERS6576310
202007856	X10	3.6.1.1.2	CipR.MSM5	*bla*$_{CTX-M-27}$, *strA, strB, aadA5, sul1, sul2, dfrA1, dfrA17, tet(A), qnrB19, mph(A), erm(B)*	S83L, D87G	S80I	ERS6576333
202008118	X10	3.6.1.1.2	CipR.MSM5	*bla*$_{CTX-M-27}$, *strA, strB, aadA5, sul1, sul2, dfrA1, dfrA17, tet(A), qnrB19, mph(A)*	S83L, D87G	S80I	ERS12446247
202008158	X10	3.6.1.1.2	CipR.MSM5	*bla*$_{CTX-M-27}$, *strA, strB, aadA5, sul1, sul2, dfrA1, dfrA17, tet(A), qnrB19, mph(A), erm(B)*	S83L, D87G	S80I	ERS6576346
202008564	X10	3.6.1.1.2	CipR.MSM5	*bla*$_{CTX-M-27}$, *strA, strB, aadA5, sul1, sul2, dfrA1, dfrA17, tet(A), qnrB19, mph(A), erm(B)*	S83L, D87G	S80I	ERS6576362
202008707	X10	3.6.1.1.2	CipR.MSM5	*bla*$_{CTX-M-27}$, *strA, strB, aadA5, sul1, sul2, dfrA1, dfrA17, tet(A), qnrB19, mph(A)*	S83L, D87G	S80I	ERS6578764
202100373	X10	3.6.1.1.2	CipR.MSM5	*bla*$_{CTX-M-27}$, *strA, strB, aadA5, sul1, sul2, dfrA1, dfrA17, tet(A), qnrB19, mph(A)*	S83L, D87G	S80I	ERS12156949
202100420	X10	3.6.1.1.2	CipR.MSM5	*bla*$_{CTX-M-27}$, *strA, strB, aadA5, sul1, sul2, dfrA1, dfrA17, tet(A), qnrB19, mph(A)*	S83L, D87G	S80I	ERS12156952
202100759	X10	3.6.1.1.2	CipR.MSM5	*bla*$_{CTX-M-27}$, *strA, strB, aadA5, sul1, sul2, dfrA1, dfrA17, tet(A), qnrB19, mph(A), erm(B)*	S83L, D87G	S80I	ERS12156964
202101412	X10	3.6.1.1.2	CipR.MSM5	*bla*$_{CTX-M-27}$, *strA, strB, aadA5, sul1, sul2, dfrA1, dfrA17, tet(A), qnrB19, mph(A), erm(B)*	S83L, D87G	S80I	ERS12156980
202101541	X10	3.6.1.1.2	CipR.MSM5	*bla*$_{CTX-M-27}$, *aadA5, sul1, dfrA1, dfrA17, qnrB19, mph(A), erm(B)*	S83L, D87G	S80I	ERS12156987
202101624	X10	3.6.1.1.2	CipR.MSM5	*bla*$_{CTX-M-27}$, *strA, strB, aadA5, sul1, sul2, dfrA1, dfrA17, tet(A), qnrB19, mph(A), erm(B)*	S83L, D87G	S80I	ERS12156996
202101651	X10	3.6.1.1.2	CipR.MSM5	*bla*$_{CTX-M-27}$, *strA, strB, aadA5, sul1, sul2, dfrA1, dfrA17, tet(A), qnrB19, mph(A), erm(B)*	S83L, D87G	S80I	ERS12156997
202101727	X10	3.6.1.1.2	CipR.MSM5	*bla*$_{CTX-M-27}$, *strA, strB, aadA5, sul1, sul2, dfrA1, dfrA17, tet(A), qnrB19, mph(A), erm(B)*	S83L, D87G	S80I	ERS12157001
202101879	X10	3.6.1.1.2	CipR.MSM5	*bla*$_{CTX-M-27}$, *strA, strB, aadA5, sul1, sul2, dfrA1, dfrA17, tet(A), qnrB19, mph(A), erm(B)*	S83L, D87G	S80I	ERS12157007
202101885	X10	3.6.1.1.2	CipR.MSM5	*bla*$_{CTX-M-27}$, *strA, strB, aadA5, sul1, sul2, dfrA1, dfrA17, tet(A), qnrB19, mph(A)*	S83L, D87G	S80I	ERS12157008
202101930	X10	3.6.1.1.2	CipR.MSM5	*bla*$_{CTX-M-27}$, *strA, strB, aadA5, sul1, sul2, dfrA1, dfrA17, tet(A), qnrB19, mph(A), erm(B)*	S83L, D87G	S80I	ERS12157011
202102119	X10	3.6.1.1.2	CipR.MSM5	*bla*$_{CTX-M-27}$, *strA, strB, aadA5, sul1, sul2, dfrA1, dfrA17, tet(A), qnrB19, mph(A), erm(B)*	S83L, D87G	S80I	ERS12157014
202102200	X10	3.6.1.1.2	CipR.MSM5	*bla*$_{CTX-M-27}$, *strA, strB, aadA5, sul1, sul2, dfrA1, dfrA17, tet(A), qnrB19, mph(A), erm(B)*	S83L, D87G	S80I	ERS12446248
202102776	X10	3.6.1.1.2	CipR.MSM5	*bla*$_{CTX-M-27}$, *strA, strB, aadA5, sul1, sul2, dfrA1, dfrA17, tet(A), qnrB19, mph(A), erm(B)*	S83L, D87G	S80I	ERS12157026
202102820	X10	3.6.1.1.2	CipR.MSM5	*bla*$_{CTX-M-27}$, *strA, strB, aadA5, sul1, sul2, dfrA1, dfrA17, tet(A), qnrB19, mph(A), erm(B)*	S83L, D87G	S80I	ERS12157027
202102980	X10	3.6.1.1.2	CipR.MSM5	*bla*$_{CTX-M-27}$, *strA, strB, aadA5, sul1, sul2, dfrA1, dfrA17, tet(A), qnrB19, mph(A)*	S83L, D87G	S80I	ERS12157031

202102993	X10	3.6.1.1.2	CipR.MSM5	*bla*$_{CTX-M-27}$, *strA, strB, aadA5, sul1, sul2, dfrA1, dfrA17, tet(A), qnrB19, mph(A), erm(B)*	S83L, D87G	S80I	ERS12157032
202103005	X10	3.6.1.1.2	CipR.MSM5	*bla*$_{CTX-M-27}$, *strA, strB, sul2, dfrA1, tet(A), qnrB19, erm(B)*	S83L, D87G	S80I	ERS12157033
202103026	X10	3.6.1.1.2	CipR.MSM5	*bla*$_{CTX-M-27}$, *strA, strB, aadA5, sul1, sul2, dfrA1, dfrA17, tet(A), qnrB19, mph(A), erm(B)*	S83L, D87G	S80I	ERS12157034
202103166	X10	3.6.1.1.2	CipR.MSM5	*bla*$_{CTX-M-27}$, *strA, strB, aadA5, sul1, sul2, dfrA1, dfrA17, tet(A), qnrB19, mph(A), erm(B)*	S83L, D87G	S80I	ERS12157040
202103248	X10	3.6.1.1.2	CipR.MSM5	*bla*$_{CTX-M-27}$, *strA, strB, aadA5, sul1, sul2, dfrA1, dfrA17, tet(A), qnrB19, mph(A), erm(B)*	S83L, D87G	S80I	ERS12157044
202103290	X10	3.6.1.1.2	CipR.MSM5	*bla*$_{CTX-M-27}$, *strA, strB, sul2, dfrA1, tet(A), qnrB19, erm(B)*	S83L, D87G	S80I	ERS12157048
202103342	X10	3.6.1.1.2	CipR.MSM5	*bla*$_{CTX-M-27}$, *strA, strB, aadA5, sul1, sul2, dfrA1, dfrA17, tet(A), qnrB19, mph(A)*	S83L, D87G	S80I	ERS12157051
202103419	X10	3.6.1.1.2	CipR.MSM5	*bla*$_{CTX-M-27}$, *strA, strB, sul2, dfrA1, tet(A), qnrB19, erm(B)*	S83L, D87G	S80I	ERS12157053
202103424	X10	3.6.1.1.2	CipR.MSM5	*bla*$_{CTX-M-27}$, *strA, strB, sul2, dfrA1, tet(A), qnrB19, erm(B)*	S83L, D87G	S80I	ERS12157055
202103567	X10	3.6.1.1.2	CipR.MSM5	*bla*$_{CTX-M-27}$, *strA, strB, aadA5, sul1, sul2, dfrA1, dfrA17, tet(A), qnrB19, mph(A), erm(B)*	S83L, D87G	S80I	ERS12157058
202103754	X10	3.6.1.1.2	CipR.MSM5	*bla*$_{CTX-M-27}$, *strA, strB, aadA5, sul1, sul2, dfrA1, dfrA17, tet(A), qnrB19, mph(A), erm(B)*	S83L, D87G	S80I	ERS12157060
202103844	X10	3.6.1.1.2	CipR.MSM5	*bla*$_{CTX-M-27}$, *strA, strB, aadA5, sul1, sul2, dfrA1, dfrA17, tet(A), qnrB19, mph(A), erm(B)*	S83L, D87G	S80I	ERS12157061
202103880	X10	3.6.1.1.2	CipR.MSM5	*bla*$_{CTX-M-27}$, *strA, strB, sul2, dfrA1, tet(A), qnrB19, erm(B)*	S83L, D87G	S80I	ERS12157062
202103930	X10	3.6.1.1.2	CipR.MSM5	*bla*$_{CTX-M-27}$, *strA, strB, aadA5, sul1, sul2, dfrA1, dfrA17, tet(A), qnrB19, mph(A), erm(B)*	S83L, D87G	S80I	ERS12157064
202104060	X10	3.6.1.1.2	CipR.MSM5	*bla*$_{CTX-M-27}$, *strA, strB, aadA5, sul1, sul2, dfrA1, dfrA17, tet(A), qnrB19, mph(A), erm(B)*	S83L, D87G	S80I	ERS12157066
202104078	X10	3.6.1.1.2	CipR.MSM5	*bla*$_{CTX-M-27}$, *strA, strB, sul2, dfrA1, tet(A), qnrB19, erm(B)*	S83L, D87G	S80I	ERS12157067
202104261	X10	3.6.1.1.2	CipR.MSM5	*bla*$_{CTX-M-27}$, *strA, strB, aadA5, sul1, sul2, dfrA1, dfrA17, tet(A), qnrB19, mph(A), erm(B)*	S83L, D87G	S80I	ERS12157068
202104281	X10	3.6.1.1.2	CipR.MSM5	*bla*$_{CTX-M-27}$, *strA, strB, aadA5, sul1, sul2, dfrA1, dfrA17, tet(A), qnrB19, mph(A), erm(B)*	S83L, D87G	S80I	ERS12157069
202104371	X10	3.6.1.1.2	CipR.MSM5	*bla*$_{CTX-M-27}$, *strA, strB, aadA5, sul1, sul2, dfrA1, dfrA17, tet(A), qnrB19, mph(A), erm(B)*	S83L, D87G	S80I	ERS12157071
202104519	X10	3.6.1.1.2	CipR.MSM5	*bla*$_{CTX-M-27}$, *strA, strB, aadA5, sul1, sul2, dfrA1, dfrA17, tet(A), qnrB19, mph(A), erm(B)*	S83L, D87G	S80I	ERS12157072
202104685	X10	3.6.1.1.2	CipR.MSM5	*bla*$_{CTX-M-27}$, *strA, strB, aadA5, sul1, sul2, dfrA1, dfrA17, tet(A), qnrB19, mph(A), erm(B)*	S83L, D87G	S80I	ERS12157074
202104831	X10	3.6.1.1.2	CipR.MSM5	*bla*$_{CTX-M-27}$, *strA, strB, sul1, sul2, dfrA1, dfrA5, tet(A), qnrB19, qnrS1, mph(A), erm(B)*	S83L, D87G	S80I	ERS12157076
202105132	X10	3.6.1.1.2	CipR.MSM5	*bla*$_{CTX-M-27}$, *strA, strB, aadA5, sul1, sul2, dfrA1, dfrA17, tet(A), qnrB19, mph(A)*	S83L, D87G	S80I	ERS12157079
202105166	X10	3.6.1.1.2	CipR.MSM5	*bla*$_{CTX-M-27}$, *strA, strB, aadA5, sul1, sul2, dfrA1, dfrA17, tet(A), qnrB19, mph(A), erm(B)*	S83L, D87G	S80I	ERS12157080
202105284	X10	3.6.1.1.2	CipR.MSM5	*bla*$_{CTX-M-27}$, *strA, strB, sul2, dfrA1, tet(A), qnrB19, erm(B)*	S83L, D87G	S80I	ERS12157083
202105285	X10	3.6.1.1.2	CipR.MSM5	*bla*$_{CTX-M-27}$, *strA, strB, sul2, dfrA1, tet(A), qnrB19, erm(B)*	S83L, D87G	S80I	ERS12157084
202105373	X10	3.6.1.1.2	CipR.MSM5	*bla*$_{CTX-M-27}$, *strA, strB, aadA5, sul1, sul2, dfrA1, dfrA17, tet(A), qnrB19, mph(A), erm(B)*	S83L, D87G	S80I	ERS12157087
202105408	X10	3.6.1.1.2	CipR.MSM5	*bla*$_{CTX-M-27}$, *strA, strB, aadA5, sul1, sul2, dfrA1, dfrA17, tet(A), qnrB19, mph(A), erm(B)*	S83L, D87G	S80I	ERS12157089
202105440	X10	3.6.1.1.2	CipR.MSM5	*bla*$_{CTX-M-27}$, *strA, strB, aadA5, sul1, sul2, dfrA1, dfrA17, tet(A), qnrB19, mph(A), erm(B)*	S83L, D87G	S80I	ERS12157092
202105446	X10	3.6.1.1.2	CipR.MSM5	*bla*$_{CTX-M-27}$, *dfrA1, qnrB19, erm(B)*	S83L, D87G	S80I	ERS12157093
202105523	X10	3.6.1.1.2	CipR.MSM5	*bla*$_{CTX-M-27}$, *strA, strB, aadA5, sul1, sul2, dfrA1, dfrA17, tet(A), mph(A), erm(B)*	S83L, D87G	S80I	ERS12157094
202105574	X10	3.6.1.1.2	CipR.MSM5	*bla*$_{CTX-M-27}$, *strA, strB, aadA5, sul1, sul2, dfrA1, dfrA17, tet(A), qnrB19, mph(A), erm(B)*	S83L, D87G	S80I	ERS12157095
202105953	X10	3.6.1.1.2	CipR.MSM5	*bla*$_{CTX-M-27}$, *strA, strB, aadA5, sul1, sul2, dfrA1, dfrA17, tet(A), qnrB19, mph(A), erm(B)*	S83L, D87G	S80I	ERS12157110
202106029	X10	3.6.1.1.2	CipR.MSM5	*bla*$_{CTX-M-27}$, *strA, strB, aadA5, sul1, sul2, dfrA1, dfrA17, tet(A), qnrB19, mph(A), erm(B)*	S83L, D87G	S80I	ERS12157114
202106219	X10	3.6.1.1.2	CipR.MSM5	*bla*$_{CTX-M-27}$, *strA, strB, aadA5, sul1, sul2, dfrA1, dfrA17, tet(A), mph(A), erm(B)*	S83L, D87G	S80I	ERS12157121
202106247	X10	3.6.1.1.2	CipR.MSM5	*bla*$_{CTX-M-27}$, *strA, strB, aadA5, sul1, sul2, dfrA1, dfrA17, tet(A), qnrB19, mph(A), erm(B)*	S83L, D87G	S80I	ERS12157122
202106455	X10	3.6.1.1.2	CipR.MSM5	*bla*$_{CTX-M-27}$, *strA, strB, aadA5, sul1, sul2, dfrA1, dfrA17, tet(A), mph(A), erm(B)*	S83L, D87G	S80I	ERS12157133
202106629	X10	3.6.1.1.2	CipR.MSM5	*bla*$_{CTX-M-27}$, *strA, strB, aadA5, sul1, sul2, dfrA1, dfrA17, tet(A), qnrB19, mph(A), erm(B)*	S83L, D87G	S80I	ERS12157142
202106664	X10	3.6.1.1.2	CipR.MSM5	*bla*$_{CTX-M-27}$, *aadA5, sul1, dfrA1, dfrA17, qnrB19, mph(A), erm(B)*	S83L, D87G	S80I	ERS12157145
202106716	X10	3.6.1.1.2	CipR.MSM5	*bla*$_{CTX-M-27}$, *strA, strB, aadA5, sul1, sul2, dfrA1, dfrA17, tet(A), qnrB19, mph(A), erm(B)*	S83L, D87G	S80I	ERS12157148
202106961	X10	3.6.1.1.2	CipR.MSM5	*bla*$_{CTX-M-27}$, *strA, strB, aadA5, sul1, sul2, dfrA1, dfrA17, tet(A), qnrB19, mph(A), erm(B)*	S83L, D87G	S80I	ERS12157163
202107022	X10	3.6.1.1.2	CipR.MSM5	*bla*$_{CTX-M-27}$, *strA, strB, aadA5, sul1, sul2, dfrA1, dfrA17, tet(A), qnrB19, mph(A), erm(B)*	S83L, D87G	S80I	ERS12157165
202107051	X10	3.6.1.1.2	CipR.MSM5	*bla*$_{CTX-M-27}$, *strA, strB, aadA5, sul1, sul2, dfrA1, dfrA17, tet(A), qnrB19, mph(A), erm(B)*	S83L, D87G	S80I	ERS12157167

202107052	X10	3.6.1.1.2	CipR.MSM5	*bla*$_{CTX-M-27}$, *strA, strB, aadA5, sul1, sul2, dfrA1, dfrA17, tet(A), qnrB19, mph(A), erm(B)*	S83L, D87G	S80I	ERS12157168
202107188	X10	3.6.1.1.2	CipR.MSM5	*bla*$_{CTX-M-27}$, *aadA5, sul1, dfrA1, dfrA17, qnrB19, mph(A), erm(B)*	S83L, D87G	S80I	ERS12157174
202107323	X10	3.6.1.1.2	CipR.MSM5	*bla*$_{CTX-M-27}$, *strA, strB, aadA5, sul1, sul2, dfrA1, dfrA17, tet(A), qnrB19, mph(A), erm(B)*	S83L, D87G	S80I	ERS12157179
202107395	X10	3.6.1.1.2	CipR.MSM5	*bla*$_{CTX-M-27}$, *strA, strB, aadA5, sul1, sul2, dfrA1, dfrA17, tet(A), mph(A)*	S83L, D87G	S80I	ERS12157182
202107415	X10	3.6.1.1.2	CipR.MSM5	*bla*$_{CTX-M-27}$, *strA, strB, aadA5, sul1, sul2, dfrA1, dfrA17, tet(A), mph(A), erm(B)*	S83L, D87G	S80I	ERS12157183
202107446	X10	3.6.1.1.2	CipR.MSM5	*bla*$_{CTX-M-27}$, *strA, strB, aadA5, sul1, sul2, dfrA1, dfrA17, tet(A), qnrB19, mph(A), erm(B)*	S83L, D87G	S80I	ERS12157184
202107466	X10	3.6.1.1.2	CipR.MSM5	*bla*$_{CTX-M-27}$, *strA, strB, aadA5, sul1, sul2, dfrA1, dfrA17, tet(A), mph(A), erm(B)*	S83L, D87G	S80I	ERS12157186
202107818	X10	3.6.1.1.2	CipR.MSM5	*bla*$_{CTX-M-27}$, *strA, strB, aadA5, sul1, sul2, dfrA1, dfrA17, tet(A), mph(A), erm(B)*	S83L, D87G	S80I	ERS12157209
202108294	X10	3.6.1.1.2	CipR.MSM5	*bla*$_{CTX-M-27}$, *strA, strB, aadA5, sul1, sul2, dfrA1, dfrA17, tet(A), qnrB19, mph(A), erm(B)*	S83L, D87G	S80I	ERS12446254
202108795	X10	3.6.1.1.2	CipR.MSM5	*bla*$_{CTX-M-27}$, *strA, strB, aadA5, sul1, sul2, dfrA1, dfrA17, tet(A), mph(A), erm(B)*	S83L, D87G	S80I	ERS12446289
202108919	X10	3.6.1.1.2	CipR.MSM5	*bla*$_{CTX-M-27}$, *strA, strB, aadA5, sul1, sul2, dfrA1, dfrA17, tet(A), mph(A), erm(B)*	S83L, D87G	S80I	ERS12446294
202108953	X10	3.6.1.1.2	CipR.MSM5	*bla*$_{CTX-M-27}$, *strA, strB, aadA5, sul1, sul2, dfrA1, dfrA17, tet(A), qnrB19, mph(A), erm(B)*	S83L, D87G	S80I	ERS12446298
202109588	X10	3.6.1.1.2	CipR.MSM5	*bla*$_{CTX-M-27}$, *strA, strB, aadA5, sul1, sul2, dfrA1, dfrA17, tet(A), mph(A), erm(B)*	S83L, D87G	S80I	ERS12446318
202109651	X10	3.6.1.1.2	CipR.MSM5	*bla*$_{CTX-M-27}$, *strA, strB, aadA5, sul1, sul2, dfrA1, dfrA17, tet(A), qnrB19, mph(A), erm(B)*	S83L, D87G	S80I	ERS12446319
202109656	X10	3.6.1.1.2	CipR.MSM5	*bla*$_{CTX-M-27}$, *strA, strB, aadA5, sul1, sul2, dfrA1, dfrA17, tet(A), qnrB19, mph(A), erm(B)*	S83L, D87G	S80I	ERS12446320
202109849	X10	3.6.1.1.2	CipR.MSM5	*bla*$_{CTX-M-27}$, *aadA5, sul1, dfrA1, dfrA17, qnrB19, mph(A), erm(B)*	S83L, D87G	S80I	ERS12446324
202109909	X10	3.6.1.1.2	CipR.MSM5	*bla*$_{CTX-M-27}$, *strA, strB, aadA5, sul1, sul2, dfrA1, dfrA17, tet(A), mph(A), erm(B)*	S83L, D87G	S80I	ERS12446330
202109960	X10	3.6.1.1.2	CipR.MSM5	*bla*$_{CTX-M-27}$, *strA, strB, aadA5, sul1, sul2, dfrA1, dfrA17, tet(A), mph(A), erm(B)*	S83L, D87G	S80I	ERS12446335
202110146	X10	3.6.1.1.2	CipR.MSM5	*bla*$_{CTX-M-27}$, *aadA5, sul1, dfrA1, dfrA17, qnrB19, mph(A), erm(B)*	S83L, D87G	S80I	ERS12446347
202110251	X10	3.6.1.1.2	CipR.MSM5	*bla*$_{CTX-M-27}$, *aadA5, sul1, dfrA1, dfrA17, qnrB19, mph(A), erm(B)*	S83L, D87G	S80I	ERS12446356
202110403	X10	3.6.1.1.2	CipR.MSM5	*bla*$_{CTX-M-27}$, *strA, strB, aadA5, sul1, sul2, dfrA1, dfrA17, tet(A), qnrB19, mph(A), erm(B)*	S83L, D87G	S80I	ERS12446360
202110415	X10	3.6.1.1.2	CipR.MSM5	*bla*$_{CTX-M-27}$, *aadA5, sul1, dfrA1, dfrA17, qnrB19, mph(A), erm(B)*	S83L, D87G	S80I	ERS12446361
202110472	X10	3.6.1.1.2	CipR.MSM5	*bla*$_{CTX-M-27}$, *strA, strB, aadA5, sul1, sul2, dfrA1, dfrA17, tet(A), qnrB19, mph(A), erm(B)*	S83L, D87G	S80I	ERS12446362
202110584	X10	3.6.1.1.2	CipR.MSM5	*bla*$_{CTX-M-27}$, *aadA5, sul1, dfrA1, dfrA17, qnrB19, mph(A), erm(B)*	S83L, D87G	S80I	ERS12446365
202110641	X10	3.6.1.1.2	CipR.MSM5	*bla*$_{CTX-M-27}$, *aadA5, sul1, dfrA1, dfrA17, qnrB19, mph(A), erm(B)*	S83L, D87G	S80I	ERS12446370
202110674	X10	3.6.1.1.2	CipR.MSM5	*bla*$_{CTX-M-27}$, *aadA5, sul1, dfrA1, dfrA17, qnrB19, mph(A), erm(B)*	S83L, D87G	S80I	ERS12446373
202110841	X10	3.6.1.1.2	CipR.MSM5	*bla*$_{CTX-M-27}$, *aadA5, sul1, dfrA1, dfrA17, qnrB19, mph(A), erm(B)*	S83L, D87G	S80I	ERS12446377
202110869	X10	3.6.1.1.2	CipR.MSM5	*bla*$_{CTX-M-27}$, *aadA5, sul1, dfrA1, dfrA17, qnrB19, mph(A), erm(B)*	S83L, D87G	S80I	ERS12446379
202110877	X10	3.6.1.1.2	CipR.MSM5	*bla*$_{CTX-M-27}$, *aadA5, sul1, dfrA1, dfrA17, qnrB19, mph(A), erm(B)*	S83L, D87G	S80I	ERS12446381
202110960	X10	3.6.1.1.2	CipR.MSM5	*bla*$_{CTX-M-27}$, *strA, strB, aadA5, sul1, sul2, dfrA1, dfrA17, tet(A), qnrB19, mph(A), erm(B)*	S83L, D87G	S80I	ERS12446386
202110961	X10	3.6.1.1.2	CipR.MSM5	*bla*$_{CTX-M-27}$, *strA, strB, aadA5, sul1, sul2, dfrA1, dfrA17, tet(A), qnrB19, mph(A), erm(B)*	S83L, D87G	S80I	ERS12446387
202111030	X10	3.6.1.1.2	CipR.MSM5	*bla*$_{CTX-M-27}$, *strA, strB, aadA5, sul1, sul2, dfrA1, dfrA17, tet(A), qnrB19, mph(A), erm(B)*	S83L, D87G	S80I	ERS12446392
202111082	X10	3.6.1.1.2	CipR.MSM5	*bla*$_{CTX-M-27}$, *strA, strB, aadA5, sul1, sul2, dfrA1, dfrA17, tet(A), qnrB19, mph(A), erm(B)*	S83L, D87G	S80I	ERS12446394
202111134	X10	3.6.1.1.2	CipR.MSM5	*bla*$_{CTX-M-27}$, *strA, strB, aadA5, sul1, sul2, dfrA1, dfrA17, tet(A), mph(A), erm(B)*	S83L, D87G	S80I	ERS12446395
202111144	X10	3.6.1.1.2	CipR.MSM5	*bla*$_{CTX-M-27}$, *strA, strB, sul2, dfrA1, tet(A), qnrB19, erm(B)*	S83L, D87G	S80I	ERS12446396
202111184	X10	3.6.1.1.2	CipR.MSM5	*bla*$_{CTX-M-27}$, *aadA5, sul1, dfrA1, dfrA17, qnrB19, mph(A), erm(B)*	S83L, D87G	S80I	ERS12446399
202200143	X10	3.6.1.1.2	CipR.MSM5	*bla*$_{CTX-M-27}$, *aadA5, sul1, dfrA1, dfrA17, qnrB19, mph(A), erm(B)*	S83L, D87G	S80I	ERS12446406
201908234	X11	3.6.1.1.2	CipR.MSM5	*bla*$_{TEM-1B}$, *bla*$_{CTX-M-3}$, *strA, strB, aadA5, sul1, sul2, dfrA1, dfrA17, tet(A), mph(A), erm(B)*	S83L, D87G	S80I	ERS12446240
202106367	X12	3.6.1	CipR-Parent	*bla*$_{CTX-M-15}$, *strA, strB, sul2, dfrA1, tet(A), qnrS1, mph(A)*	S83L	-	ERS12157130
202108535	X12	3.6.1	CipR-Parent	*bla*$_{CTX-M-15}$, *strA, strB, sul2, dfrA1, tet(A), qnrB19, qnrS1, mph(A)*	S83L	-	ERS12446269
202102843	X13	3.6.1.1.2	CipR.MSM5	*bla*$_{TEM-1B}$, *bla*$_{CTX-M-15}$, *strA, strB, aadA5, sul1, sul2, dfrA1, dfrA17, tet(A), qnrS1, mph(A), erm(B)*	S83L, D87G	S80I	ERS12157029

QRDR, quinolone resistance-determining region

Supplementary Table 3. Description of the AMR elements identified after long-read sequencing

Genotype	XDR cluster	Isolate	QRDR mutations		Chromosomal AMR genes[#]	AMR plasmids					GenBank accession nos.
			gyrA	*parC*		Name	Size in kb	Inc type[£]	PTU type[£]	AMR genes	
**3.6.1_CipR-parent**											
	X12	202106367	S83L		*dfrA1*	p202106367-3	87,521	FII	FE	*bla*$_{\text{CTX-M-15}}$, *mph(A)*, *qnrS1*	OP038295
						p202106367-6	8,379	NT	NT	*strA*, *strB*, *sul2*, *tet(A)*	OP038296
		202108535	S83L		*dfrA1*	p202108535-3	87,540	FII	FE	*bla*$_{\text{CTX-M-15}}$, *mph(A)*, *qnrS1*	OP038298
						p202108535-7	8,379	NT	NT	*strA*, *strB*, *sul2*, *tet(A)*	OP038299
						p202108535-13	2,699	Col(pH)	E62	*qnrB19*	OP038297
**3.6.1.1_CipR**											
	X1	201701093	S83L	S80I	*bla*$_{\text{CTX-M-15}}$, *dfrA1*	p201701093-2	102,387	B/O/K/Z	B/O/K/Z	*sul1*, *dfrA5*, *mph(A)*, *erm(B)*, *qnrS13*	OP038271
	X5	201703316	S83L, D87G	S80I	*dfrA1*	p201703316-2	82,941	FII	FE	*bla*$_{\text{CTX-M-15}}$, *aadA5*, *sul1*, *dfrA17*, *mph(A)*, *qnrS1*	OP038274
	X6	201908033	S83L, D87G	S80I	*bla*$_{\text{CTX-M-15}}$, *dfrA1*	p201908033-3	73,493	FII	FE	*mph(A)*, *erm(B)*	OP038279
						p201908033-4	8,401	NT	E63	*strA*, *strB*, *sul2*, *tet(A)*	OP038280
**3.6.1.1.1_CipR.SEA**											
	X2	201903956	S83L, D87G	S80I	*dfrA1*	p201903956-2	78,123	FII	FE	*bla*$_{\text{CTX-M-27}}$, *aadA5*, *sul1*, *dfrA17*, *mph(A)*	OP038278
						p201903956-4	8,401	NT	E63	*strA*, *strB*, *sul2*, *tet(A)*	OP038303
	X3	201502874	S83L, D87G	S80I	*dfrA1*	p201502874-3	79,375	FII	FE	*bla*$_{\text{CTX-M-27}}$, *aadA5*, *sul1*, *dfrA17*, *mph(A)*	OP038267
						p201502874-5	8,401	NT	NT	*strA*, *strB*, *sul2*, *tet(A)*	OP038268
	X4	201603898	S83L, D87G	S80I	*dfrA1*	p201603898-2	102,642	B/O/K/Z	B/O/K/Z	*bla*$_{\text{CTX-M-55}}$, *aac(3)-IIa*, *mph(A)*	OP038269
						p201603898-4	8,401	NT	NT	*strA*, *strB*, *sul2*, *tet(A)*	OP038270
		201702782	S83L, D87G	S80I	*dfrA1*	p201702782-2	97,029	B/O/K/Z	B/O/K/Z	*bla*$_{\text{CTX-M-55}}$, *aac(3)-IIa*, *mph(A)*	OP038272
						p201702782-3	8,401	NT	E63	*strA*, *strB*, *sul2*, *tet(A)*	OP038273
**3.6.1.1.2_CipR.MSM5**											
	X7	201605743	S83L, D87G	S80I	*dfrA1*	p201605743-3	84,796	I1	I1	*bla*$_{\text{CTX-M-3}}$	OP038300
						p201605743-4	80,127	FII	FE	*bla*$_{\text{TEM-1B}}$, *aadA5*, *sul1*, *dfrA17*, *mph(A)*, *erm(B)*	OP038301
	X8	201809101	S83L, D87G	S80I	*dfrA1*	p201809101-4	106,936	I1	I1	*bla*$_{\text{CTX-M-15}}$, *aadA1*, *sul2*, *dfrA1*, *qnrS1*	OP038275
						p201809101-5*	88,949	B/O/K/Z	B/O/K/Z	*sul2*, *tet(B)*	OP038276
						p201809101-6	80,134	FII	FE	*bla*$_{\text{TEM-1B}}$, *aadA5*, *sul1*, *dfrA17*, *mph(A)*, *erm(B)*	OP038277
	X9	202000562	S83L, D87G	S80I	*dfrA1*	p202000562-4	76,702	FII	FE	*bla*$_{\text{CTX-M-134}}$, *strA*, *sul2*, *mph(A)*	OP038284
						p202000562-5	8,379	NT	NT	*strA*, *strB*, *sul2*, *tet(A)*	OP038285
	X10	202008118	S83L, D87G	S80I	*dfrA1*	p202008118-4	78,102	FII	FE	*bla*$_{\text{CTX-M-27}}$, *aadA5*, *sul1*, *dfrA17*, *mph(A)*	OP038287
						p202008118-7	8,390	NT	NT	*strA*, *strB*, *sul2*, *tet(A)*	OP038288
						p202008118-16	2,579	Col(pH)	E76	*qnrB19*	OP038286

	202008564	S83L, D87G	S80I	*dfrA1*	p202008564-6	83,397	FII	FE	$bla_{CTX-M-27}$, *aadA5*, *sul1*, *dfrA17*, *mph(A)*, *erm(B)*	OP038290
					p202008564-7	8,390	NT	NT	*strA*, *strB*, *sul2*, *tet(A)*	OP038291
					p202008564-21	2,579	Col(pH)	E76	*qnrB19*	OP038289
X11	201908234	S83L, D87G	S80I	*dfrA1*	p201908234-4	86,101	I1	I1	$bla_{CTX-M-3}$	OP038281
					p201908234-5	82,851	FII	FE	bla_{TEM-1B}, *aadA5*, *sul1*, *dfrA17*, *mph(A)*, *erm(B)*	OP038282
					p201908234-6	8,379	NT	E63	*strA*, *strB*, *sul2*, *tet(A)*	OP038283
X13	202102843	S83L, D87G	S80I	*dfrA1*	p202102843-3	89,779	I1	I1	$bla_{CTX-M-15}$, *qnrS1*	OP038292
					p202102843-4	80,145	FII	FE	bla_{TEM-1B}, *aadA5*, *sul1*, *dfrA17*, *mph(A)*, *erm(B)*	OP038293
					p202102843-8	8,401	NT	E63	*strA*, *strB*, *sul2*, *tet(A)*	OP038294

[#], The *dfrA1* gene is a class 2 integron gene cassette present in a chromosomally inserted Tn*7* (downstream from the bacterial gene *glmS*); *, non-circularized; [£], NT, non typable, Col(pH) means Col(pHAD28).

Supplementary Table 4. List of the 69 XDR (or inferred to be XDR) *S. sonnei* isolates published by other groups

Isolate	Year	Country[§]	Genotype	CTX-M allele	Accession number	Reference
03_0044	2014	Vietnam	3.6.1.1.1	CTX-M-55	ERR3094145	5, 6
02_1181	2014	Vietnam	3.6.1.1.1	CTX-M-15	SRR3474168	5, 6
01_0643	2015	Vietnam	3.6.1.1.1	CTX-M-55	ERR3094113	5
01_0839	2015	Vietnam	3.6.1.1.1	CTX-M-55	ERR3094114	5
03_0194	2015	Vietnam	3.6.1.1.1	CTX-M-14	ERR3094152	5, 6
03_0268	2015	Vietnam	3.6.1.1.1	CTX-M-55	ERR3094155	5
03_0514	2015	Vietnam	3.6.1.1.1	CTX-M-14	ERR3094167	5
03_0780	2015	Vietnam	3.6.1.1.1	CTX-M-14	ERR3094172	5
187596	2015	United Kingdom	3.6.1.1.1	CTX-M-14	SRR3530603	6
01_0932	2016	Vietnam	3.6.1.1.1	CTX-M-55	ERR3094115	5
01_0953	2016	Vietnam	3.6.1.1.1	CTX-M-55	ERR3094117	5
01_0975	2016	Vietnam	3.6.1.1.1	CTX-M-55	ERR3094118	5
01_1008	2016	Vietnam	3.6.1.1.1	CTX-M-55	ERR3094120	5
01_1077	2016	Vietnam	3.6.1.1.1	CTX-M-55	ERR3094122	5
02_2142	2016	Vietnam	3.6.1.1.1	CTX-M-55	ERR3094143	5
249376	2016	United Kingdom	3.6.1.1	CTX-M-15	SRR5017524	6
261400	2016	United Kingdom	3.6.1.1.1	CTX-M-55	SRR7186960	6
AUSMDU00005736	2016	Australia	3.6.1.1.1	CTX-M-55	SRR7886021	6, 7, 8
AUSMDU00010002	2016	Australia	3.6.1.1.1	CTX-M-55	SRR7886023	6, 7, 8
PNUSAE006498	2017	USA	3.6.1.1.1	CTX-M-14	SRR5434942	6
PNUSAE006476	2017	USA	3.6.1.1	CTX-M-15	SRR5434950	6
PNUSAE008493	2017	USA	3.6.1.1.1	CTX-M-55	SRR5875679	6
408647	2017	United Kingdom	3.6.1.1	CTX-M-15	SRR7244238	6
381252	2017	United Kingdom	3.6.1.1	CTX-M-15	SRR7358007	6
AUSMDU00006624	2017	Australia	3.6.1.1.1	CTX-M-55	SRR7886280	6, 7, 8
AUSMDU00016501	2018	Australia	3.6.1.1.2	CTX-M-55	SRR10506699	6, 7
AUSMDU00016500	2018	Australia	3.6.1.1.2	CTX-M-55	SRR10506700	6, 7
AUSMDU00016499	2018	Australia	3.6.1.1.2	CTX-M-55	SRR10506702	6, 7
506788	2018	United Kingdom	3.6.1.1.2	CTX-M-15	SRR7221227	6
533443	2018	United Kingdom	3.6.1.1.1	CTX-M-55	SRR7286389	6
493395	2018	United Kingdom	3.6.1.1.2	CTX-M-15	SRR7291660	6
549526	2018	United Kingdom	3.6.1.1	CTX-M-15	SRR7367460	6
579964	2018	United Kingdom	3.6.1.1.2	CTX-M-15	SRR7826920	6
AUSMDU00013452	2018	Australia	3.6.1.1.2	CTX-M-55	SRR7885960	6, 7, 8
AUSMDU00012898	2018	Australia	3.6.1.1.2	CTX-M-55	SRR7886243	6, 7, 8
627378	2018	United Kingdom	3.6.1.1.2	CTX-M-55	SRR8117834	6
658645	2018	United Kingdom	3.6.1.1	CTX-M-15	SRR8380048	6
3123885	2019	Switzerland	3.6.1.1.2	CTX-M-134	CP049163, CP049164	4
19-0820-1561	2019	Switzerland	3.6.1.1	CTX-M-15	CP049173, CP049173	4
IBESS186	2019	Netherlands	3.6.1.1	CTX-M-15	ERR3330659	6
IBESS245	2019	Netherlands	3.6.1.1	CTX-M-15	ERR3330799	6
790576	2019	United Kingdom	3.6.1.1	CTX-M-15	SRR10005842	6
795372	2019	United Kingdom	3.6.1.1	CTX-M-27	SRR10037531	6
801162	2019	United Kingdom	3.6.1.1	CTX-M-27	SRR10088450	6
819800	2019	United Kingdom	3.6.1.1.2	CTX-M-15	SRR10271372	6
821179	2019	United Kingdom	3.6.1.1	CTX-M-15	SRR10302327	6
824423	2019	United Kingdom	3.6.1.1	CTX-M-15	SRR10314892	6
AUSMDU00027242	2019	Australia	3.6.1.1.2	CTX-M-14	SRR10506633	6, 7
AUSMDU00025310	2019	Australia	3.6.1.1.2	CTX-M-14	SRR10506731	6, 7, 9
AUSMDU00027777	2019	Australia	3.6.1.1.2	CTX-M-14	SRR10506734	6, 7, 9
AUSMDU00028447	2019	Australia	3.6.1.1.2	CTX-M-14	SRR10506844	6, 7, 9
AUSMDU00028554	2019	Australia	3.6.1.1	CTX-M-15	SRR10506849	6, 7, 9
845732	2019	United Kingdom	3.6.1.1.2	CTX-M-134	SRR10559611	6
866406	2019	United Kingdom	3.6.1.1	CTX-M-15	SRR10858744	6
AUSMDU00038900	2019	Australia	3.6.1.1	CTX-M-15	SRR12131989	9

AUSMDU00032220	2019	Australia	3.6.3	CTX-M-15	SRR12131999	9
AUSMDU00032223	2019	Australia	3.6.3	CTX-M-15	SRR12132002	9
738605	2019	United Kingdom	3.6.1.1	CTX-M-15	SRR9050419	6
AUSMDU00040532	2020	Australia	3.6.1.1.2	CTX-M-27	SRR12132000	9
AUSMDU00040564	2020	Australia	3.6.1.1.2	CTX-M-27	SRR12132003	9
1324211	2022	United Kingdom	3.6.1.1.2	CTX-M-27	SRR15512411	10
1502196	2022	United Kingdom	3.6.1.1.2	CTX-M-27	SRR16612616	10
1519721	2022	United Kingdom	3.6.1.1.2	CTX-M-27	SRR16842843	10
1519755	2022	United Kingdom	3.6.1.1.2	CTX-M-27	SRR16842826	10
1527837	2022	United Kingdom	3.6.1.1.2	CTX-M-27	SRR16940973	10
1527854	2022	United Kingdom	3.6.1.1.2	CTX-M-27	SRR16940985	10
1534306	2022	United Kingdom	3.6.1.1.2	CTX-M-27	SRR17038739	10
1538007	2022	United Kingdom	3.6.1.1.2	CTX-M-27	SRR17099017	10
1538717	2022	United Kingdom	3.6.1.1.2	CTX-M-27	SRR17120129	10

[§] Travel to Southeast Asia for isolates AUSMDU00005736 and AUSMDU00010002; travel to Nepal for isolate 19-0820-1561 and travel to Israel for isolate 3123885.

Supplementary References

1. Worley, J.N. et al. Genomic drivers of multidrug-Resistant *Shigella* affecting vulnerable patient populations in the United States and abroad. *mBio* **12**, e03188-20 (2021).

2. Locke, R.K., Greig, D.R., Jenkins, C., Dallman, T.J. & Cowley, L.A. Acquisition and loss of CTX-M plasmids in *Shigella* species associated with MSM transmission in the UK. *Microb. Genom.* **7**, 000644 (2021).

3. Baker, K.S. et al. Intercontinental dissemination of azithromycin-resistant shigellosis through sexual transmission: a cross-sectional study. *Lancet Infect. Dis.* **15**, 913–921 (2015).

4. Campos-Madueno, E.I. et al. Rapid increase of CTX-M-producing *Shigella sonnei* isolates in Switzerland due to spread of common plasmids and international clones. *Antimicrob. Agents Chemother.* **64**, e01057-20 (2020).

5. Thanh Duy, P. et al. Commensal *Escherichia coli* are a reservoir for the transfer of XDR plasmids into epidemic fluoroquinolone-resistant *Shigella sonnei*. *Nat. Microbiol.* **5,** 256-64 (2020).

6. Hawkey, J. et al. Global population structure and genotyping framework for genomic surveillance of the major dysentery pathogen, *Shigella sonnei*. *Nat. Commun.* **12**, 2684 (2021).

7. Williamson, D., Ingle, D. & Howden, B. Extensively Drug-Resistant Shigellosis in Australia among Men Who Have Sex with Men. *N. Engl. J. Med.* **381**, 2477–9 (2019).

8. Ingle, D.J. et al. Co-circulation of multidrug-resistant *Shigella* among men who have sex with men in Australia. *Clin. Infect. Dis.* **69**, 1535–1544 (2019).

9. Ingle, D.J. et al. Prolonged outbreak of multidrug-resistant *Shigella sonnei* harboring $bla_{\text{CTX-M-27}}$ in Victoria, Australia. *Antimicrob. Agents Chemother.* **64**, e01518-20 (2020).

10. Charles, H. et al. Outbreak of sexually transmitted, extensively drug-resistant *Shigella sonnei* in the UK, 2021-22: a descriptive epidemiological study. *Lancet Infect. Dis.* **22,** 1503-1510 (2022).
