# Supplementary material for: Rapid emergence of extensively drug-resistant Shigella sonnei in France
Source: Nat Commun. 2023 Jan 28;14:462. doi: 10.1038/s41467-023-36222-8 (PMC9883819; doi:10.1038/s41467-023-36222-8)
Supplement: Supplementary file 3 — Description of Additional Supplementary Files [file 41467_2023_36222_MOESM3_ESM.pdf]

**Description of Additional Supplementary Files**

File Name: Supplementary Data 1

Description: List of the 3,140 *S. sonnei* genomic sequences from isolates and historical strains of the FNRC-ESS included in the phylogenetic tree. The following are shown: isolate name, alternative isolate name, year of isolation, country of isolation, isolated in Mainland France or in Overseas France, notion of international travel, EBI-ENA accession numbers.
