# Supplementary material for: Rapid emergence of extensively drug-resistant Shigella sonnei in France
Source: Nat Commun. 2023 Jan 28;14:462. doi: 10.1038/s41467-023-36222-8 (PMC9883819; doi:10.1038/s41467-023-36222-8)
Supplement: Supplementary file 5 — Reporting Summary [file 41467_2023_36222_MOESM5_ESM.pdf]

nature portfolio

Corresponding author(s): François-Xavier WEILL

Last updated by author(s): 01/13/2023

Reporting Summary

Nature Portfolio wishes to improve the reproducibility of the work that we publish. This form provides structure for consistency and transparency in reporting. For further information on Nature Portfolio policies, see our Editorial Policies and the Editorial Policy Checklist.

Statistics

For all statistical analyses, confirm that the following items are present in the figure legend, table legend, main text, or Methods section.

n/a	Confirmed	
☐	☒	The exact sample size (*n*) for each experimental group/condition, given as a discrete number and unit of measurement
☒	☐	A statement on whether measurements were taken from distinct samples or whether the same sample was measured repeatedly
☐	☒	The statistical test(s) used AND whether they are one- or two-sided<br>*Only common tests should be described solely by name; describe more complex techniques in the Methods section.*
☒	☐	A description of all covariates tested
☒	☐	A description of any assumptions or corrections, such as tests of normality and adjustment for multiple comparisons
☒	☐	A full description of the statistical parameters including central tendency (e.g. means) or other basic estimates (e.g. regression coefficient) AND variation (e.g. standard deviation) or associated estimates of uncertainty (e.g. confidence intervals)
☐	☒	For null hypothesis testing, the test statistic (e.g. *F*, *t*, *r*) with confidence intervals, effect sizes, degrees of freedom and *P* value noted<br>*Give P values as exact values whenever suitable.*
☒	☐	For Bayesian analysis, information on the choice of priors and Markov chain Monte Carlo settings
☒	☐	For hierarchical and complex designs, identification of the appropriate level for tests and full reporting of outcomes
☒	☐	Estimates of effect sizes (e.g. Cohen's *d*, Pearson's *r*), indicating how they were calculated

Our web collection on statistics for biologists contains articles on many of the points above.

Software and code

Policy information about availability of computer code

Data collection	Excel version 15.41
Data analysis	EnteroBase (http://enterobase.warwick.ac.uk/), FqCleanER version 3.0 (https://gitlab.pasteur.fr/GIPhy/fqCleanER), ENA https://www.ebi.ac.uk/ena/, Sonneityping/Mykrobe version 0.9.0 (https://github.com/katholt/sonneityping), Snippy v4.6/BWA-MEM version 0.7.17/ Freebayes version 1.3.2 (https://github.com/tseemann/snippy), Gubbins version 3.2.0 (https://github.com/sanger-pathogens/gubbins), RAxML version 8.2.12 (https://github.com/stamatak/standard-RAxML), iTOL version 6 (https://itol.embl.de), ResFinder version 4.0.1 https://cge.cbs.dtu.dk/services/ResFinder/),BLAST version 2.2.26 (http://www.ncbi.nlm.nih.gov/BLAST/), UniCycler version 0.4.8 (https://github.com/rrwick/Unicycler), Pilon version 1.23 (https://github.com/broadinstitute/pilon/releases/), Prokka version 1.14.5 (https://github.com/tseemann/prokka), BRIG version 0.95 (http://sourceforge.net/projects/brig), PlasmidFinder version 2.1.1. (https://cge.cbs.dtu.dk/services/PlasmidFinder/), pMLST version 1.2 (https://cge.cbs.dtu.dk/services/pMLST/), COPLA version 1.0 (https://castillo.dicom.unican.es/copla/), SPAdes version 3.9.0 (https://cab.spbu.ru/software/spades/).

For manuscripts utilizing custom algorithms or software that are central to the research but not yet described in published literature, software must be made available to editors and reviewers. We strongly encourage code deposition in a community repository (e.g. GitHub). See the Nature Portfolio guidelines for submitting code & software for further information.

Data

Policy information about availability of data

All manuscripts must include a data availability statement. This statement should provide the following information, where applicable:

- Accession codes, unique identifiers, or web links for publicly available datasets
- A description of any restrictions on data availability
- For clinical datasets or third party data, please ensure that the statement adheres to our policy

The publicly available sequences used in this study are available in GenBank under accession numbers CP053763, NC_016822, AE014073, NZ_MW396858, LN624486, CP049176, CP049164, CP049174, CP049186, CP053751. Short-read sequence data generated in this study were submitted to EnteroBase (https://enterobase.warwick.ac.uk/) and to the European Nucleotide Archive (ENA, https://www.ebi.ac.uk/ena/) under study number PRJEB44801. Whole-genome assemblies have been deposited in FigShare (https://doi.org/10.6084/m9.figshare.21594033.v1). All the accession numbers of the short-read sequences produced and used in this study are listed in Supplementary Table 2 and Supplementary Data 1. The plasmid sequences obtained were deposited in GenBank (https://www.ncbi.nlm.nih.gov/genbank/) under accession numbers OP038267-OP038301 and OP038303 (Supplementary Table 3).

Human research participants

Policy information about studies involving human research participants and Sex and Gender in Research.

Reporting on sex and gender	No human research participant (only use of basic metadata present in the notification form accompanying bacterial isolates, please see methods).
Population characteristics	No human research participant.
Recruitment	No human research participant.
Ethics oversight	No human research participant.

Note that full information on the approval of the study protocol must also be provided in the manuscript.

Field-specific reporting

Please select the one below that is the best fit for your research. If you are not sure, read the appropriate sections before making your selection.

☐ Life sciences ☐ Behavioural & social sciences ☒ Ecological, evolutionary & environmental sciences

For a reference copy of the document with all sections, see nature.com/documents/nr-reporting-summary-flat.pdf

Ecological, evolutionary & environmental sciences study design

All studies must disclose on these points even when the disclosure is negative.

Study description	The goal of this study was to identify and characterize extensively drug-resistant (i.e., simultaneously resistant to ciprofloxacin, third-generation cephalosporins and azithromycin) Shigella sonnei isolates in France between 2005 and 2021.
Research sample	Our sample consists of all the 7,121 Shigella sonnei isolates (only one per patient) received and confirmed at the French National Reference Center for E. coli, Shigella and Salmonella (NRC-ESS), Institut Pasteur, between 2005 and 2021. It has been estimated that the NRC-ESS surveillance system detects 50-60% of laboratory-confirmed Shigella infections in France.
Sampling strategy	Antimicrobial susceptibility testing was performed on all the 7,121 isolates. Whole-genome sequencing was used on all the 164 XDR isolates detected by antimicrobial susceptibility testing. We placed them into a larger phylogenetic context by including other routine genomes to reach a total of 3,109 (from the 7,121 S. sonnei isolates collected between 2005 and 2021). Most of these genomes were collected between 2017 (the year in which genomic surveillance began in France) and 2021. During that time frame, we received 2,618 S. sonnei isolates and included the 2,558 genomes (97.7%) that passed the EnteroBase quality control criteria. From 2005 to 2016 , our selection comprised 551 genomes, in particular, 96.7% (89/92) of all isolates resistant to third-generation cephalosporins (3GCs), 56.3% (294/522) of all isolates resistant to ciprofloxacin (CIP), and 96.7% (116/120) of the isolates resistant to azithromycin (AZM) (detected from April 2014). No statistical methods were used to determine the number of strains isolated between 2005 and 2017 to be sequenced. We tried to sequence all isolates resistant to 3GCs and AZM (some isolates could not be located or did not grow) and 50% of isolates resistant to CIP (due to budget constraints). However, the proportion of selected isolates for each type of antibiotic resistance ranging from 56.3% to 96.7% was estimed to be sufficient to provide a representativity of these resistances. We also included 31 published genomes from our collection (1945-2004), to enrich the dataset with rare lineages of S. sonnei (L1, L2 and L4), and the S. sonnei reference genome 53G (Holt et al. Nat Genet 2012).
Data collection	The data were collected on an excel spreadsheet by S.L. and E.N.
Timing and spatial scale	Our study period was from January 1st 2005 (when routine antimicrobial susceptibility testing started) to December 31th 2021 (the

Timing and spatial scale	last full year before manuscript submission). We used all isolates received on a regular, routine basis, from mainland France and its overseas territories in South America (French Guiana), the Caribbean (Martinique, Guadeloupe) and the Indian Ocean (La Réunion, Mayotte).
Data exclusions	In case of multiple isolates for a same patient, we only included the first one. For the genomic analysis, we only included the genomic sequences that passed the quality control criteria of EnteroBase (https://enterobase.warwick.ac.uk/species/index/ecoli). Hence, between 2017 and 2021, 60 of the 2,618 produced genomes were excluded.
Reproducibility	For all the 164 XDR strains, we ensured phenotypic and genomic concordance for antimicrobial resistance. The phylogenomic analysis was performed thrice with two different reference genomes as outgroups. Antimicrobial susceptibility testing (AST) was performed only once according to standard clinical and public health microbiology procedures. if the AST results were not concordant with the antibiotic resistance gene content inferred from the genomic analysis, both AST and genomic sequencing were redone until final concordance.
Randomization	The 164 XDR S. sonnei strains were not allocated into different experimental groups (observational study). Therefore, randomization was not used in this study.
Blinding	For all experiments (antimicrobial susceptibility testing, genomic analysis of antibiotic resistance determinants and phylogenetic analysis), only the isolate name was used as identifier. Therefore we were "blind" to the associated metadata before comparative analysis.

Did the study involve field work? ☐ Yes ☒ No

Reporting for specific materials, systems and methods

We require information from authors about some types of materials, experimental systems and methods used in many studies. Here, indicate whether each material, system or method listed is relevant to your study. If you are not sure if a list item applies to your research, read the appropriate section before selecting a response.

Materials & experimental systems

n/a	Involved in the study
☒	☐ Antibodies
☒	☐ Eukaryotic cell lines
☒	☐ Palaeontology and archaeology
☒	☐ Animals and other organisms
☒	☐ Clinical data
☒	☐ Dual use research of concern

Methods

n/a	Involved in the study
☒	☐ ChIP-seq
☒	☐ Flow cytometry
☒	☐ MRI-based neuroimaging
